# Biocontrol and growth promotion potential of *Bacillus velezensis* NT35 on *Panax ginseng* based on the multifunctional effect

**DOI:** 10.3389/fmicb.2024.1447488

**Published:** 2024-07-30

**Authors:** Xueqing Li, Jiarui Wang, Hang Shen, Chenxi Xing, Lingxin Kong, Yu Song, Wanpeng Hou, Jie Gao, Yun Jiang, Changqing Chen

**Affiliations:** ^1^College of Plant Protection, Jilin Agricultural University, Changchun, China; ^2^College of Life Science, Jilin Agricultural University, Changchun, China; ^3^Jilin Shenwang Plant Protection Co., Ltd., Fusong, China

**Keywords:** *Bacillus velezensis*, *Panax ginseng*, biocontrol, plant growth promotion, multifunctional mechanisms

## Abstract

The *Bacillus velezensis* strain NT35, which has strong biocontrol ability, was isolated from the rhizosphere soil of *Panax ginseng*. The antifungal effects of the NT35 strain against the mycelium and spore growth of *Ilyonectria robusta*, which causes ginseng rusty root rot, were determined. The inhibitory rate of *I. robusta* mycelial growth was 94.12% when the concentration of the NT35 strain was 10^7^ CFU·mL^−1^, and the inhibitory rates of *I. robusta* sporulation and spore germination reached 100 and 90.31%, respectively, when the concentration of the NT35 strain was 10^4^ and 10^8^ CFU·mL^−1^, respectively. Strain NT35 had good prevention effects against ginseng rust rot indoors and in the field with the control effect 51.99%, which was similar to that of commercial chemical and biocontrol agents. The labeled strain NT35-Rif^160^-Stre^400^ was obtained and colonized ginseng roots, leaves, stems and rhizosphere soil after 90 days. *Bacillus velezensis* NT35 can induce a significant increase in the expression of five defensive enzyme-encoding genes and ginsenoside biosynthesis-related genes in ginseng. In the rhizosphere soil, the four soil enzymes and the microbial community improved during different periods of ginseng growth in response to the biocontrol strain NT35. The NT35 strain can recruit several beneficial bacteria, such as *Luteimonas*, *Nocardioides*, *Sphingomonas*, and *Gemmatimonas*, from the rhizosphere soil and reduce the relative abundance of *Ilyonectria*, *Fusarium*, *Neonectria* and *Dactylonectria*, which cause root rot and rusty root rot in ginseng plants. The disease indices were significantly negatively correlated with the abundances of *Sphingomonas* and *Trichoderma*. Additionally, *Sphingomonadales*, *Sphingomonadaceae* and actinomycetes were significantly enriched under the NT35 treatment according to LEfSe analysis. These results lay the foundation for the development of a biological agent based on strain NT35.

## Highlights

- The strain NT35 has ability to control rusty root rot and promote growth of ginseng.
- Increasing gene expression of defense enzymes and ginsenoside biosynthesis of ginseng.
- To colonize and significantly improve microbial community in rhizosphere soil of ginseng.

## 1 Introduction

*Panax ginseng*, one of the most well-known Chinese herbal medicinal plants, is now grown in the northeastern region of China (You et al., 2022). Rusty root rot disease caused by *Ilyonectria robusta* is the most common and serious root disease that occurs during *P, ginseng* cultivation in China (Lee et al., 2015; Farh et al., 2018). At present the general incidence of ginseng rusty root rot in China is 20%–30%, and more seriously, some more than 70%. The quality and yield of ginseng were severely affected, resulting in huge property losses (Wang et al., 2019; Guan et al., 2020). For a long time, this disease has been controlled mainly by chemical fungicides. However, chemical control is not ideal for treating soil-borne diseases in perennial plants. Additionally, prolonged use of chemical fungicides results in pathogen resistance, residue risk, soil microecological imbalance and environmental pollution (Wołejko et al., 2020).

Biocontrol can reduce or prevent the occurrence of plant diseases and has the benefits of being environmentally friendly, safe, and highly efficient (Sharma and Manhas, 2022). For instance, *Bacillus amyloliquefaciens* was reported to be effective at controlling *Phytophthora capsica* (Bhusal and Mmbaga, 2020). Fu et al. (2010) reported the biocontrol potential of *B. subtilis* against banana leaf spot caused by *Pseudocercospora musae* and postharvest anthracnose caused by *Colletotrichum musae*. The *B. velezensis* strain B19 can control Fusarium root rot in *Panax notoginseng* (Wang C. X. et al., 2023). Thus, biocontrol by beneficial microorganisms may be chosen as an alternative means of reducing ginseng rusty root rot without negatively impacting the environment compared to chemical control with fungicides. Previous studies have shown that the rhizosphere acts as an important bridge between plants and soil for the exchange of substances through plant roots and helps establish mutual relationships (Ahkami et al., 2017). The bacteria in rhizospheric soil are responsible for many important ecological functions, such as disease control (Linden et al., 2022) and resistance induction (Zhang et al., 2022). System induction resistance is an important mechanism for plants to fight pathogens. *Bacillus velezensis* effective against *Botrytis cinerea* by inducing systemic resistance in tomato (Stoll et al., 2021). And biocontrol bacteria have been reported to improve rhizosphere soil microecology by recruiting beneficial microorganisms, reducing the abundance of harmful communities, and increasing microbial communities diversity (Zhao et al., 2022). Using rhizospheric soil bacteria has been a good strategy for the efficient biocontrol of soil-borne diseases. The objective of this study was to determine the ability of the *B. velezensis* strain NT35 to inhibit *I. robusta* growth and control ginseng rusty root rot via *in vitro* and field experiments. The expression of defense enzyme-encoding genes in ginseng induced by the biocontrol bacterium was also analyzed. The high-throughput sequence technique was used to study the microbial community structure, which may improve rhizosphere soil microecology through the strain NT35. The results lay a foundation for the development and application of the biocontrol bacterium NT35 on ginseng plants.

## 2 Materials and methods

### 2.1 Strains and culture

The *B. velezensis* strain NT35 was previously isolated from the rhizosphere soil of *P. ginseng* in Ji’an, Jilin Province, China, and the eight pathogens causing ginseng diseases including *I. robusta*, *Sclerotinia schinseng*, *Phytophthora cactorum*, *Rhizoctonia solani*, *Alternaria panax*, *Botrytis cinerea*, *Colletotrichum panacicola*, *Fusarium solani* were obtained was obtained from the Laboratory of Integrated Management of Plant Diseases, Jilin Agricultural University, Changchun, Jilin Province, China, and stored at −80°C. The plant pathogens were incubated on potato dextrose agar (PDA) at 25°C for 5 days. *Bacillus velezensis* NT35 was cultured on lysogeny broth agar medium (LB) and incubated at 28°C for 2 days in dark.

### 2.2 Inhibition of *Ilyonectria robusta* mycelial growth by strain NT35

A dual culture assay was used to evaluate the ability of *B. velezensis* NT35 against *I. robusta* CBLJ-3 (Wang J. et al., 2023). The concentrations of *B. velezensis* NT35 were 10^9^, 10^8^, 10^7^, 10^6^, 10^5^, 10^4^, 10^3^, 10^2^, and 10^1^ CFU·mL^−1^. In the control, only the *I. robusta* CBLJ-3 mycelium plug was inoculated, and sterile agar was added to the Petri dishes; three replicates were used for each treatment. Then, the plates were incubated at 25°C for 7 days. Measurements were calculated by the formula I (%) = [(C − T)/C] × 100 (I: percentage of inhibition, C: growth of pathogen in control plate, T: growth of pathogen in treatment plate). Additionally, the antifungal efficacy of the NT35 strain against 14 other plant pathogens was determined by the confrontation culture method.

The effect of *B. velezensis* NT35 on the mycelial weight of *I. robusta* was evaluated as described below (Wang C. X. et al., 2023). An 8 mm diameter of the fungal agar plug of *I. robusta* CBLJ-3 was inoculated into 90 mL of PD fluid in a 250 mL flask and cultured at 150 rpm and 28°C for 2 days, after which 10 mL of 1 × 10^8^ CFU/mL NT35 fermentation broth was added. The mixture was further cultured with shaking at 25°C, and the mycelia of *I. robusta* were sampled at 1, 2, 3, 4, 5, 6, and 7 dpi after inoculation with *B. velezensis* NT35. The mycelial samples were filtered and washed three times with sterile water. The mycelial dry weight of each treatment was determined after drying at 80°C for 24 h. Three replicates were performed for each group.

### 2.3 Inhibition of *Ilyonectria robusta* spore production and germination by the NT35 strain

The hemocytometer method was used to determine the inhibitory effect of the NT35 strain on the spore production and germination of *I. robusta* (Wang J. et al., 2023). The strain CBLJ-3 was inoculated on plates mixed with serially diluted fermentation broth as described above and cultured at 25°C. The conidia were flushed with 5 mL of sterile water after the colonies reached more than 60 mm in diameter, and the plates were mixed with sterile water as a control. The conidia yield was determined using a hemocytometer, and the inhibition rate was calculated as follows: inhibition rate (%) = 100 × (control conidia production − treatment conidia production)/control conidia production. When the CBLJ-3 spore germination rate of the control group reached greater than 90%, the germinated conidia were counted using a hemocytometer, and the inhibition rate was calculated as follows: inhibition rate (%) = 100 × (control germination rate − treatment germination rate)/control germination rate. Each treatment was replicated three times.

### 2.4 *In vivo* effect of the NT35 strain on inhibiting *Ilyonectria robusta* growth in ginseng roots

An *in vitro* inhibition test was also performed in ginseng roots according to Wang C. X. et al. (2023). The three-year-old healthy ginseng roots were disinfected with 2% sodium hypochlorite solution for 90 s, rinsed twice with sterile distilled water and dried at room temperature for 2 h. A total of 10 μL of 1 × 10^8^ CFU·mL^−1^ NT35 fermentation broth was inoculated at each site, at which three wound sites with a depth of 3 mm were generated on each ginseng root by using a sterile pipette tip with a diameter of 2 mm. The four treatments included the following: (A) water as a negative control; (B) *I. robusta* as a positive control; (C) Prevention treatment: inoculation with NT35 for 1 day and then with *I. robusta*; and (D) Therapeutic treatment: inoculation with *I. robusta* for 1 day and then with NT35. All the inoculated plants were incubated at 25°C.

### 2.5 Growth-promoting effect of strain NT35 on ginseng plants

The Salkowski method was used to screen for high IAA production by the strain NT35 (Ait Bessai et al., 2022). The phosphate solubilization ability of the strains was determined by cultivation on Pikovskaya’s agar medium and by the Mo-Sb colorimetric method (Liu et al., 2021). Potassium solubilization was tested by using Aleksandrow’s agar medium and a flame atomic absorption spectrometer (Iyer et al., 2017). The nitrogen fixation potency was determined after the strain NT35 was inoculated in ASHBY nitrogen-free agar medium and incubated at 30°C for 24 h (Xu et al., 2001). Siderophore production was determined by the CAS method (Delaporte-Quintana et al., 2020), and ACC deaminase activity was determined by the Honma method (Tiwari et al., 2016).

The NT35 strain was inoculated in LB liquid, cultured at 160 rpm at 28°C for 12 h and subsequently centrifuged at 10,000 × g for 5 min. The collected cells were dissolved in distilled water, and the original cells were diluted to 1 × 10^8^ CFU·mL^−1^. Ginseng seeds were first sterilized by soaking in 70% ethanol for 5 min and then in 0.5% sodium hypochlorite for 3 min and rinsed three times with distilled water. These disinfected seeds were immersed in the above bacterial suspension of strain NT35 for 4 h. Seeds treated with distilled water served as the control. All the seeds were then transferred to plates containing wetted sand (10 seeds per plate) and incubated at 25°C for 10–15 days (Jiang et al., 2022).

### 2.6 Colonization ability of strain NT35 in *Panax ginseng* and rhizosphere soil

Preparation of rifampicin solutions (2 × 10^4^ μg·mL^−1^) and streptomycin solutions (5 × 10^4^ μg·mL^−1^), The NT35 strain was activated by LB medium and incubated at 28°C and 160 rpm for 12 h. Rifampicin was uniformly mixed into the LB solid medium to make a plate containing rifampicin at a concentration of 40 μg·mL^−1^, and 0.1 mL of NT35 bacterial solution was coated on the plate, and the plate was incubated at 28°C for 3–4 days. Single colonies with the same or similar morphology as that of the original NT35 were picked for streaking and incubation. After incubation for 2–3 days, the single colonies were streaked and inoculated onto LB medium containing rifampicin at a concentration of 80 μg·mL^−1^, and antibiotic-resistant mutant strains with good growth and the same colony morphology as that of the original bacterium were always selected to obtain the mutant NT35-Rif^160^, which was able to tolerate rifampicin at a concentration of 160 μg·mL^−1^. The concentration of streptomycin of NT35 was increased step by step according to the same method from 50 μg·mL^−1^ to 400 μg·mL^−1^ to obtain the double-resistant strain NT35-Rif^160^-Stre^400^, which has the same colony characteristics as the original strain and stable drug resistance (Cohen et al., 1989). Ginseng roots were inoculated with the NT35-Rif^160^-Stre^400^ strain at 10^8^ CFU/mL, and 50 mL of liquid was added to each root. The ginseng roots, stems, leaves and rhizosphere soil were sampled after inoculation for 3, 7, 15, 30, 45, 60, or 90 days, and each treatment was repeated three times. The colonization of various parts of ginseng and soil by the NT35-Rif^160^-Stre^400^ strain was determined via the plate colony counting method. Each treatment was repeated three times.

### 2.7 qRT–PCR analysis of defense enzyme-encoding gene expression

After leaf spreading, three-year-old healthy ginseng plants were inoculated with a bacterial suspension of *B. velezensis* NT35 at a concentration of 1 × 10^8^ CFU·mL^−1^ in pots in the greenhouse, and water was inoculated as the control. The ginseng roots were sampled at 1, 3, 5, 7, 9 and 11 days after treatment and were soaked in liquid nitrogen. The total RNA of ginseng roots was extracted using the RNAiso Plus method. Reverse transcription was conducted using M-MLV reverse transcriptase (TaKaRa Biotechnology Co., Ltd., Dalian, China). The qRT-PCR assay was performed on a fluorescent quantitative PCR instrument LC96 (Roche) using Fast SYBR Mixture (Company) for qRT-PCR reactions. The 10 μL PCR mixture contained 1 μL of cDNA, 0.2 μL each of 10 μmol/L upstream and downstream primers, 5 μL of 2 × Fast SYBR mixture, 0.2 μL of 50 × Low ROX and 3.4 μL of ddH_2_O. The qRT–PCR conditions were 95°C predenaturation for 10 min, 95°C denaturation for 10 s, 60°C annealing and extension for 30 s, for a total of 40 cycles. Five genes, phenylalanine ammonia-lyase (*PAL*), β-1.3 glucanase (*β-1,3-GLU*), chitinase (*CHI*), superoxide dismutase (*SOD*) and peroxidase (*POD*), of NT35-treated ginseng plants were analyzed by using qRT–PCR (Jiang et al., 2022). Each gene was conducted with three biological and technical replicates. The relative gene expression was analyzed using the 2^−△△Ct^ method (Livak and Schmittgen, 2001; Kim et al., 2019).

### 2.8 Efficacy of strain NT35 for controlling rusty root rot in ginseng in the field

The field experiment was carried out in 2021 in Choushui township, Fusong county, Jilin Province, China. The control effect of 1 × 10^8^ cfu/g NT35 WP (Jilin Agricultural University) was evaluated in a field where ginseng rusty root rot occurs frequently. The ginseng field was divided into different plots of 1.5 m^2^ following a completely random design on 25th April. The soil of all plots was treated by mixing 8 g/m^2^ NT35 WP with commercial agents, including 8 g/m^2^ 3 × 10^11^ cfu/g YIWEI WP (Shandong Jingqing Agriculture Science and Technology Co., China), 8 g/m^2^ 2 × 10^11^ cfu/g ZNLK WP (Zhongnong Lvkang Biotechnology Co., China) and 5 g/m^2^ 50% carbendazim WP (Jiangsu Wansheng International Chemical Group, China), with no agent serving as a control. Two-year-old ginseng plants of uniform size were planted at a 7 cm depth in the soil and treated consistently. The row spacing was 20 cm, the plant spacing was 10 cm, and there were approximately 60 plants in each plot and the three random plots were sampled. All ginseng roots were harvested at the maturity stage, and disease severity was investigated (Rahman and Punja, 2005).

### 2.9 Effect of strain NT35 on soil enzyme activity

After the ginseng roots were inoculated with the NT35 bacterial suspension at a concentration of 1 × 10^8^ CFU·mL^−1^, rhizosphere soil samples were collected at four different periods of ginseng growth: leaf spread stage (13 June), fruit stage (15 July), root expansion stage (16 August), and mature stage (15 September). The soil urease activity was determined by using the method described by Kandeler and Gerber (1988). The catalase activity was tested according to Lúcia et al. (2016). The alkaline phosphatase activity was determined using the protocol described by Rahul and Anita (2016). The invertase activity was measured using the method described by Sun et al. (2021). Three replicates were carried out for each treatment.

### 2.10 qRT–PCR analysis of ginsenoside synthesis-related gene expression

Three-year-old healthy ginseng roots were inoculated with an NT35 bacterial suspension at a concentration of 1 × 10^8^ CFU·mL^−1^, and water was used as a control. Total RNA was extracted from ginseng roots using the RNAiso Plus method. Reverse transcription was conducted using M-MLV reverse transcriptase (TaKaRa Biotechnology Co., Ltd., Dalian, China). The three genes encoding squalene synthase, squalene epoxidase, and dammarediol synthase were analyzed by qRT–PCR as described above, and the *β-actin* gene was used as the internal reference. The primers used were synthesized by Sangon Biotech (Shanghai) Co., Ltd., in China (Supplementary Table S1). Each gene was conducted with three biological and technical replicates.

### 2.11 Effects of strain NT35 on the rhizosphere microbial community of ginseng

#### 2.11.1 Soil samples

The rhizosphere soil samples and sampling method for this experiment were consistent with those described in section 2.8 above. The soil microbial community was analyzed during three periods: leaf spreading, fruiting and root expansion. Three samples were taken as one replicate, and a total of 18 soil samples were obtained. Soil samples with no added agent were used as a control group.

#### 2.11.2 DNA extraction, sequencing, and data analysis

The total DNA of the soil samples was extracted by the CTAB method (Niemi et al., 2001). The V3–V4 regions of the bacterial 16S rRNA gene (Zhang et al., 2018) and the fungal ITS2 gene (Huang J. et al., 2022) were amplified. Library construction and sequence analysis were performed on an Illumina NovaSeq 6000 platform by Novogene Co., Ltd. (Beijing, China). Spearman correlation was used to analyze the relationships between soil enzymes, disease indices and the microbial community.

#### 2.11.3 Sequence processing and data analysis

After the chimeric sequences were removed, the operational taxonomic units (OTUs) were clustered at 97% sequence identity using UPARSE (Uparse v7.0.1001, http://www.drive5.com/uparse/; Edgar, 2013). The taxonomic identities of the microbiome were determined using the SSUrRNA database of Silva138^1^ (Quast et al., 2013). The alpha diversity and abundances of the OTUs were calculated between the different treatments. PCoA dimensionality reduction was performed to analyze the differences in community structure between the groups at different stages. Spearman correlation was used to study the relationships among soil enzymes, disease indices and microbial communities. Pairwise correlation and significance *p*-values were obtained.

### 2.12 Statistical analysis

Three replicates were analyzed for each treatment. The significant differences between means were calculated using Tukey’s multiple range test, followed by one-way ANOVA (^*^p < 0.05, ^**^p < 0.01, ^***^p < 0.001). For each trial, the results were subjected to analysis of variance (ANOVA), and treatment means were compared by Fisher’s protected least significant difference (LSD) test at 5% probability. Before the ANOVA, the normality of the distribution of residuals and homogeneity of variance were verified. The statistical analyzes were performed with SPSS 26.0 (Ning et al., 2022).

## 3 Results

### 3.1 Effects of strain NT35 on the growth of *Ilyonectria robusta* and other pathogens in *Panax ginseng*

All nine concentrations of *B. velezensis* NT35 inhibited the hyphal growth of *I. robusta* with different efficacies. Among the concentrations, the inhibition rate of the treatment with a concentration greater than 10^7^ CFU·mL^−1^ was more than 94.12%, which was significantly greater than that of the other treatments, and the highest inhibition rate was observed for the treatment with 10^9^ CFU·mL^−1^, which reached 99.51% (Supplementary Table S2).

**Figure 1:**
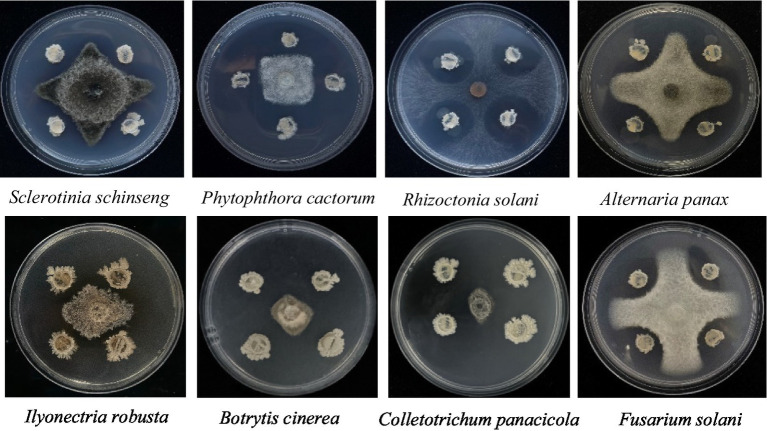
Inhibitory effect of NT35 on eight pathogenic fungi of *Panax ginseng*.

The NT35 strain not only inhibited *I. robusta* but also had a broad-spectrum inhibitory effect on seven other plant pathogens, including *Sclerotinia sclerotiorum*, *Phytophthora cactorum*, *Alternaria panax*, *Rhizoctonia solani*, *Fusarium solani*, *Botrytis cinerea*, and *Colletotrichum panacicola* (Figure 1).

The weight of *I. robusta* mycelia also decreased 7 days after inoculation with NT35 (Supplementary Figure S1). At 3 dpi and 4 dpi, there were significantly greater inhibition rates against *I. robusta* than at the other time points, and the highest rate reached 77.21% at 4 dpi. The NT35 strain was found to have a good inhibitory effect on the amount of sporulation of *I. robusta* (Supplementary Table S3). The inhibition rates exceeded 90% at all concentrations greater than 10^1^ CFU·mL^−1^. When the concentration of *B. velezensis* NT35 was greater than 10^4^ CFU·mL^−1^, the sporulation inhibition rate was 100%. Moreover, when the spore germination rate of the control group reached 91.4%, the spore germination rates of *I. robusta* treated with different concentrations of NT35 decreased. With increasing concentration, the inhibition rate increased, and the highest inhibition rate reached 93.28% at 10^9^ CFU·mL^−1^, indicating that *B. velezensis* NT35 has a good inhibitory effect on *I. robusta* sporulation and spore germination.

### 3.2 *In vivo* effect of the NT35 strain on inhibiting *Ilyonectria robusta* growth in ginseng roots

**Figure 2:**
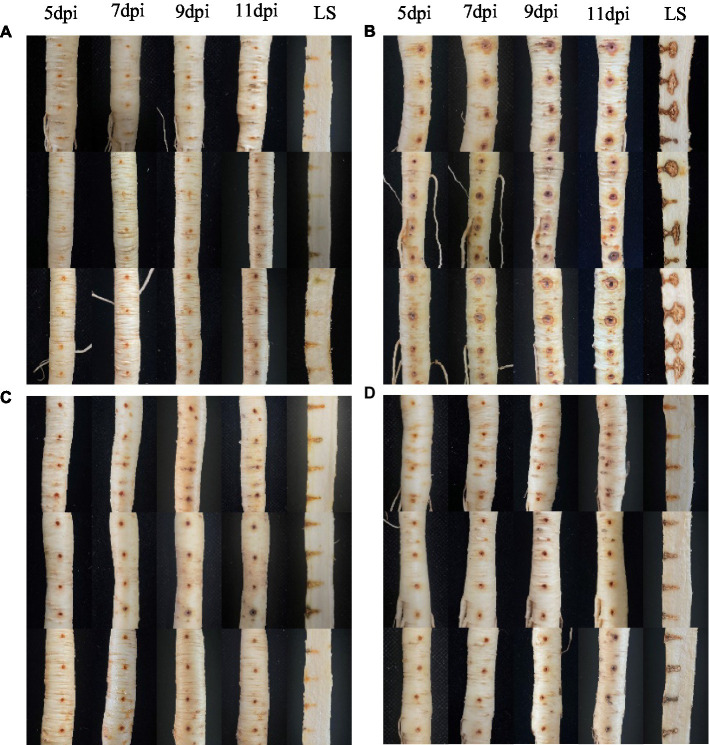
*In vivo* effect of strain NT35 inhibiting *Ilyonectria robusta* on ginseng root. LS, Longitudinal section; **(A)** Water treatment; **(B)** *I. robusta* CBLJ-3; **(C)** Prevention treatment: inoculation with NT35 for 1 day and then with *I. robusta*; **(D)** Therapeutic treatment: inoculation with *I. robusta* for 1 day and then with NT35.

The positive control with only pathogen inoculation (Figure 2B) showed typical discolouration around the inoculation site on the fifth day after inoculation with *I. robusta*. No significant discolouration was observed in the preventive treatment (Figure 2C) or the therapeutic treatment (Figure 2D) with strain NT35. The ginseng in water treatment (Figure 2A) showed only slight oxidation of the wound and its interior after inoculation, with no ginseng rusty root rot symptoms, which proves that the ginseng lesions in this experiment could only be caused by inoculation with CBLJ-3. With increasing days after inoculation, the disease spots in the pathogen-positive control gradually became larger, darker and more decomposed. However, there was no obvious deterioration in the ginseng roots after the NT35 treatment. On the 11th day after inoculation, the ginseng roots were dissected lengthwise, and the extent and area invaded by the pathogen *I. robusta* inside the roots were significantly lower than those of the control plants. The preventive treatment (Figure 2C) had the greatest effect.

### 3.3 Growth-promoting effect of strain NT35 on ginseng plants

**Figure 3:**
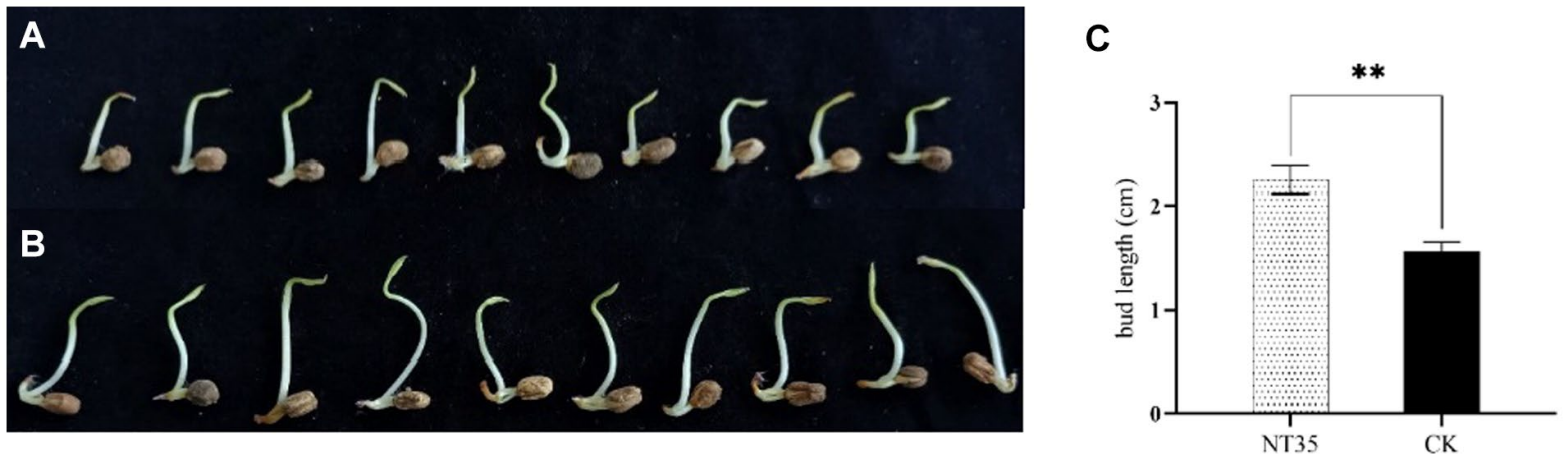
The promoting effect of strain NT35 on ginseng seed germination. **(A)** Water control; **(B)** NT35 treatment; **(C)** Seed bud length (*n* = 3 independent replicates). *p*-values were indicated by * symbol: ^**^p < 0.01; ^*^p < 0.05.

The four indicators of growth-promoting abilities of strain NT35 were determined in this study (Supplementary Figure S2). The NT35 strain has the potential for nitrogen fixation and siderophore activity, but it cannot solubilize phosphorus or potassium. The levels of IAA and ACC deaminase produced by strain NT35 were 1.392 and 0.0197 μmoL/mg·h, respectively. After 15 days, the average bud length of the ginseng plants soaked in water was 1.56 cm, while that of the plants in the NT35 treatment was 2.25 cm, which was significantly (1.44 times) greater than that of the water control. The results showed that the bacterium NT35 could promote ginseng seed germination (Figure 3).

### 3.4 Colonization of strain NT35 in ginseng and its rhizosphere soil

**Figure 4:**
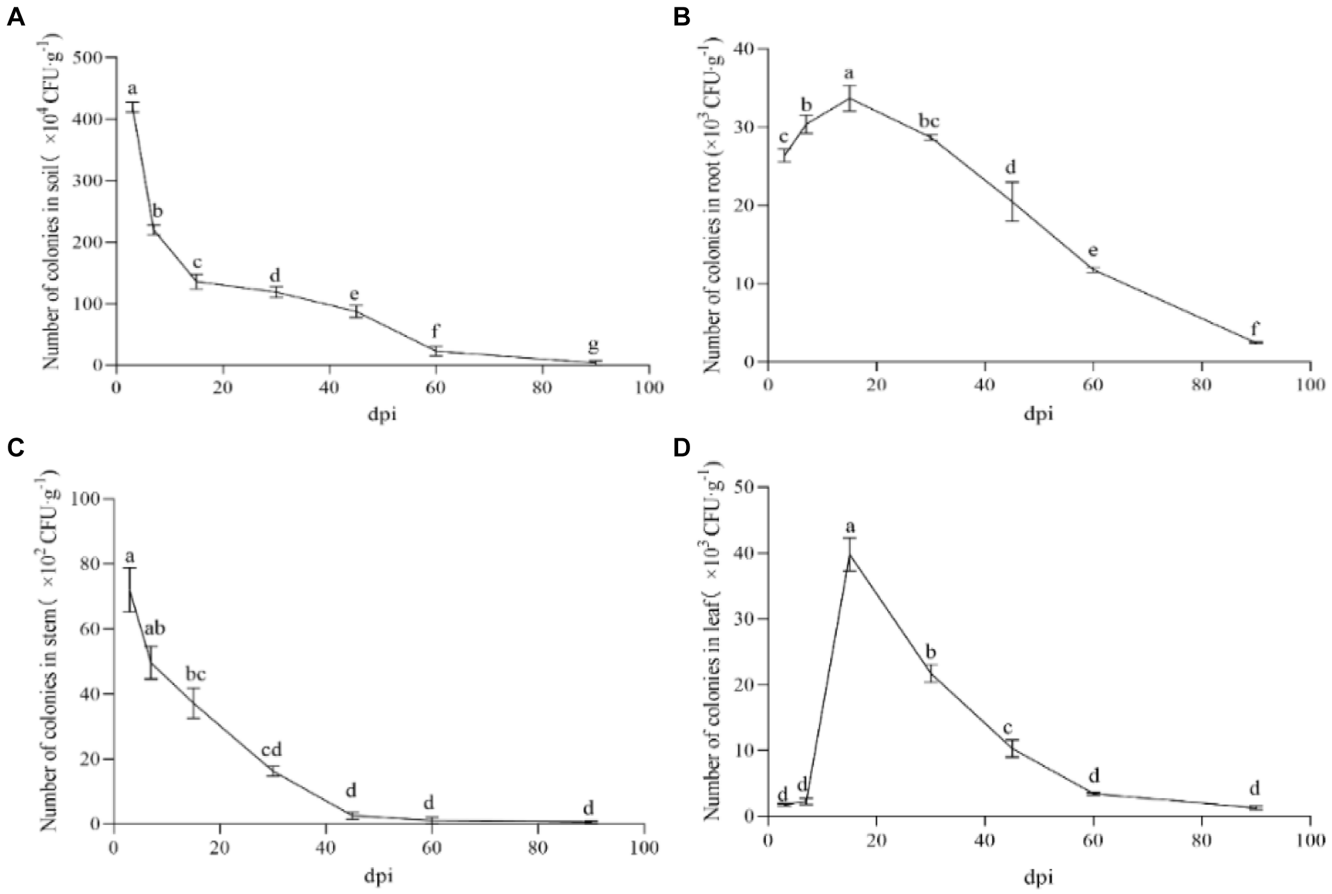
Colonization of strain NT35 in ginseng and rhizosphere soil. **(A)** rhizosphere soil; **(B)** ginseng root; **(C)** ginseng stem; **(D)** ginseng leave. *n* = 3; different values (a, b, c, d, e, f, g) between different dpi are significantly different (*p* < 0.05).

The labeled strain NT35-Rif^160^-Stre^400^ was obtained, and its ability to colonize ginseng roots, leaves, stems and rhizosphere soil was maintained for 90 days (Figure 4). In the ginseng roots and leaves, the trend first increased and then decreased, while it gradually decreased in the rhizosphere soil and stems. The colonization of NT35 in rhizosphere soil was greater than that in the roots, stems and leaves of ginseng. On the 3rd day after inoculation, the colonization level of the NT35-Rif^160^-Stre^400^ population was 4.20 × 10^6^ CFU/g in rhizosphere soil, 2.64 × 10^4^ CFU/g in roots, 7.20 × 10^3^ CFU/g in stems, and 1.77 × 10^3^ CFU/g in leaves. At 15 days after inoculation, the colonization population in the ginseng roots and leaves peaked. Up to 90 days after inoculation, the colonization amounts in the rhizosphere soil, roots, stems and leaves were 3.7× 10^4^, 2.46 × 10^3^, 0.67× 10^2^, and 1.29× 10^3^ CFU/g, respectively.

### 3.5 Expression of the defense enzyme-encoding gene induced by *Bacillus velezensis* NT35

**Figure 5:**
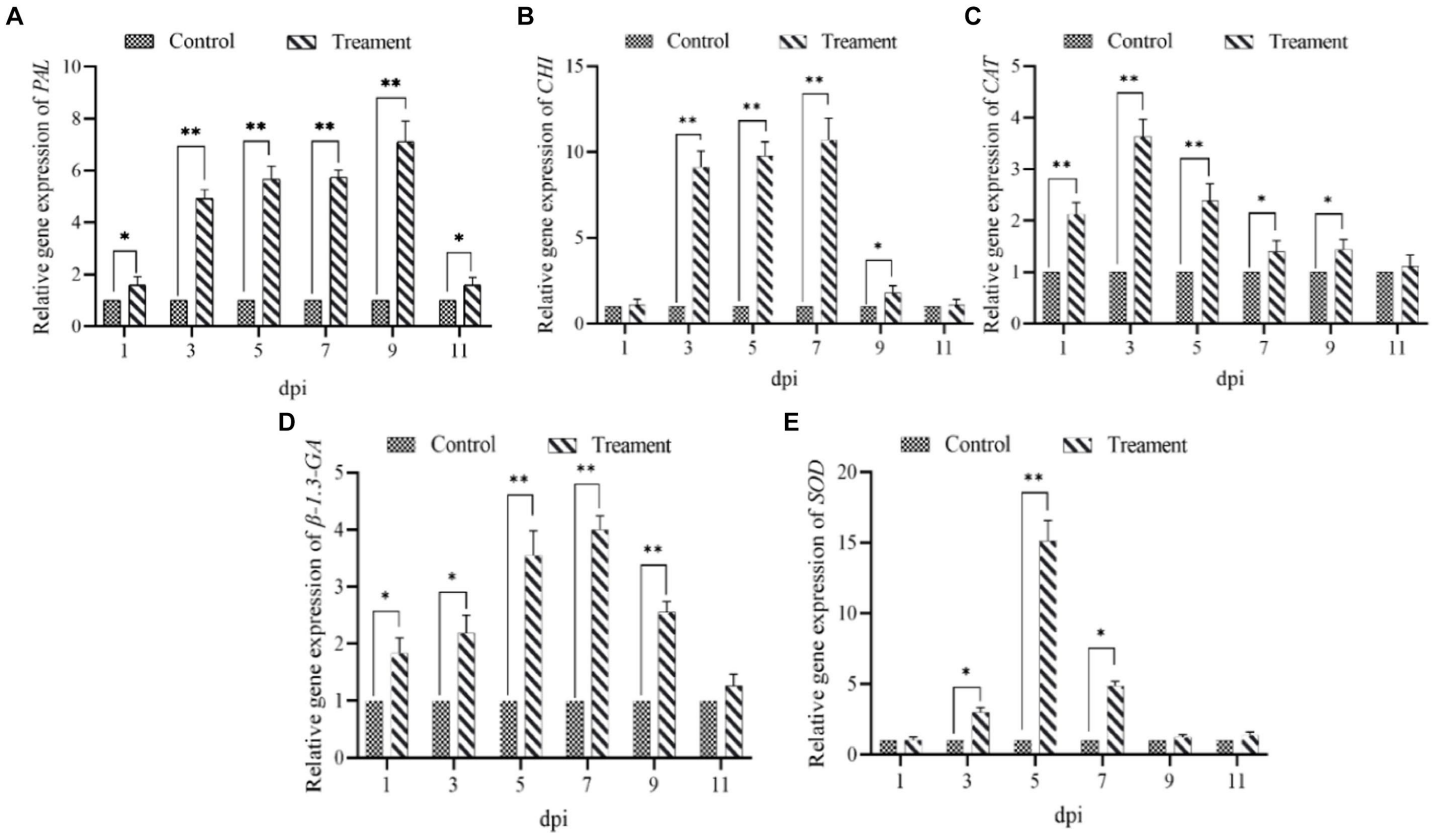
Relative expression levels of five defense genes of ginseng **(A)** PAL; **(B)** CHI; **(C)** CAT; **(D)** β-1.3-GA; **(E)** SOD.

Similarly, the expression of all five defense genes (*β-1.3-GA*, *CHI*, *PAL*, *SOD* and *CAT*) in ginseng roots inoculated with *B. velezensis* NT35 tended to increase compared to that in the control (Figure 5). The transcript expression of the *β-1.3-GA* and *CAT* genes significantly increased in response to treatment with strain NT35 from 1 to 9 dpi and peaked at 3 and 7 dpi, respectively (Figures 5C,D). *CHI* and *SOD* were significantly expressed from 3 to 9 dpi, with peaks occurring at 7 and 5 dpi, respectively (Figures 5B,E). The *PAL* genes were significantly upregulated throughout the sampling period and peaked at 9 dpi (Figure 5A). In addition, the expression of all five genes showed a decreasing trend from 9 to 11 dpi.

### 3.6 Control effect of strain NT35 on rusty root rot in ginseng in the field

In the field experiment, the disease index of the NT35 WP treatment (2.333) was lower than that of the negative control (4.859), and the control effect reached 51.99% in the NT35 WP treatment, which was not significant different from the control effects of the YIWEI WP and ZNLK biological agents and carbendazim chemical fungicide (Supplementary Figure S3). For yield, the weight of ginseng roots (191.16 g) treated with NT35 WP was significantly greater than that of those treated with the ZNLK agent or water control.

### 3.7 Effect of strain NT35 on ginseng soil enzyme activity

In general, the soil enzyme activity in ginseng plants treated with strain NT35 increased to some degree during different growth stages compared with that in plants treated with water. However, the change trend of the activity of each enzyme was different. The change in invertase activity showed a trend of increasing first and then decreasing overall, with the maximum value occurring at the fruit stage (Supplementary Figure S4A). The phosphatase activity increased continuously and peaked during the mature period of ginseng. The maximum phosphatase activity after NT35 treatment was 1988.94 μg g^−1^, which was significantly greater than that in the water control and YIWEI treatment groups (Supplementary Figure S4B). After the NT35 treatment, the soil urease activity first increased and then decreased. During the fruiting period, a maximum value of 572.26 μg/g was reached, which was almost twice as high as that of the Yiwei treatment (Supplementary Figure S4C). The maximum value of catalase activity was reached during the root expansion period and was significantly greater than that in the YIWEI treatment and water control groups (Supplementary Figure S4D).

### 3.8 Effect of NT35 on the expression of ginsenoside biosynthesis-related genes

**Figure 6:**
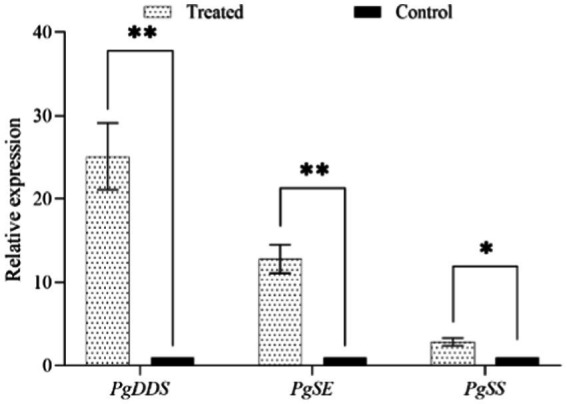
Effect of strain NT35 on relative expression of ginsenoside synthesis genes. Values represent mean ± standard deviation (*n* = 3), *p*-values were indicated by * symbol: ^**^p < 0.01; ^*^p < 0.05.

After NT35 treatment, the expression of ginsenoside biosynthesis-related genes was upregulated. The relative expression levels of the squalene synthase gene (*PgSS*), squalene epoxidase gene (*PgSE*) and dammarenediol synthase gene (*PgDDS*) in response to treatment with strain NT35 were 25.11, 12.82, and 2.85 times greater than those in response to treatment with water, respectively. The relative expression of *PgDDS* and *PgSS* in the NT35 treatment group was extremely significantly greater than that in the water control group, and the relative expression of *PgSE* was significantly greater than that in the water control group (Figure 6).

### 3.9 Effect of strain NT35 on the rhizosphere microbial community of *Panax ginseng*

#### 3.9.1 Alpha diversity and PCoA analysis

During the four stages of ginseng growth, the Shannon, Simpson, Chao1 and ACE indices of the bacterial and fungal communities changed to different degrees. The Simpson and Shannon indices of the soil bacterial and fungal communities treated with strain NT35 were significantly greater than those of the control at the flowering and root-expanding stages and significantly lower than those of the control at the leaf-spreading stage (Supplementary Figures S5A,B,E,F). The Chao1 and ACE indices of the bacterial and fungal communities were not significantly different among the three stages throughout the growth cycles (Supplementary Figures S5C,D,G,H).

According to the PCoA analysis based on the Bray–Curtis distance, the diversity of the soil microbial communities changed in response to the antagonistic bacteria NT35 treatment, as reflected by the coordinate axes. The bacterial communities produced by the *B. velezensis* NT35 treatment and the water control treatment were significantly different on the horizontal axis at the fruiting and root expansion stages, and the fungal communities at the fruiting stage were significantly different on both the horizontal and vertical axes (Supplementary Figures S6A,B).

#### 3.9.2 Phylum- and genus-level composition of the microbial community

**Figure 7:**
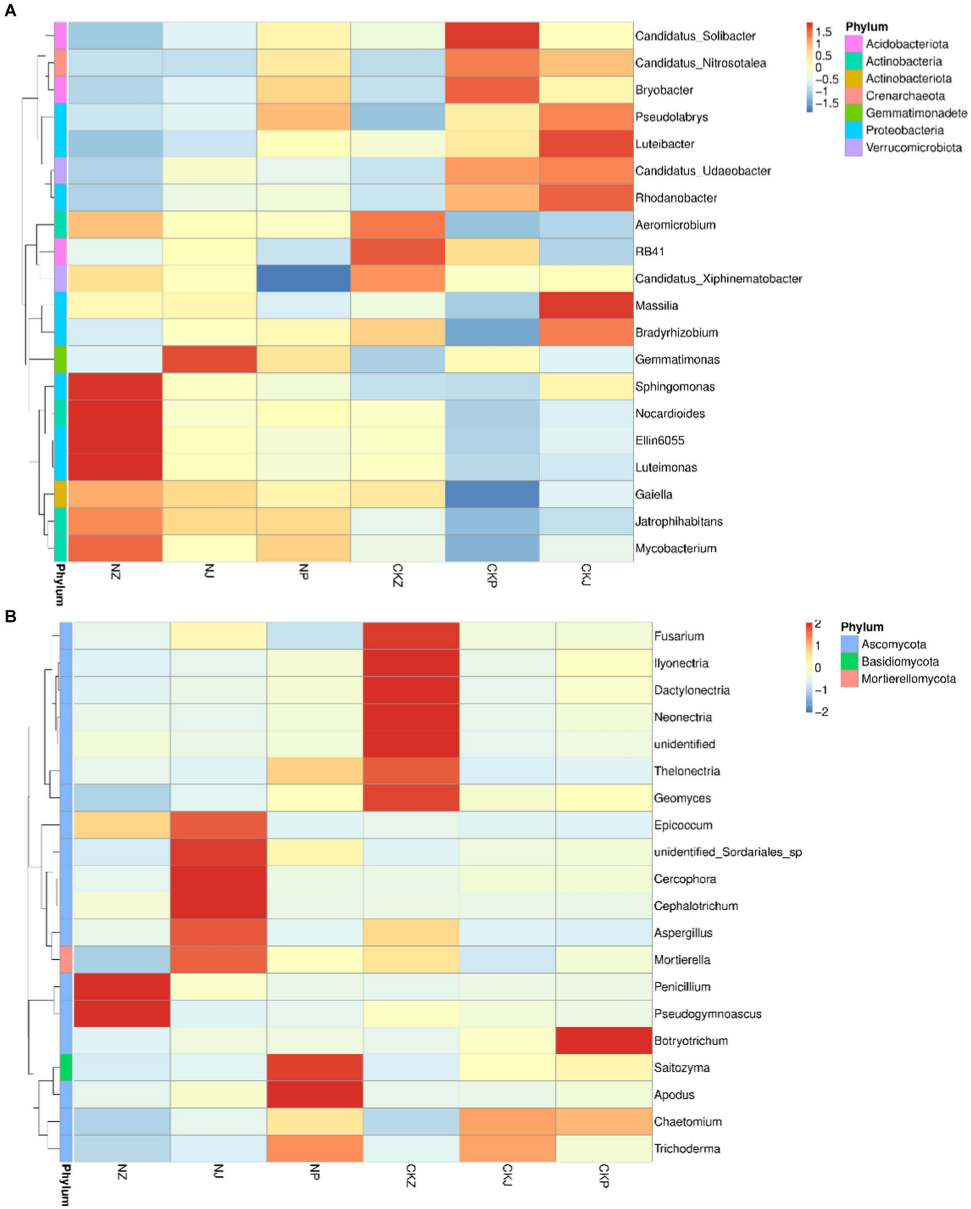
Composition of bacteria and fungi at different ginseng growth stages. **(A)** Bacterial community composition; **(B)** Fungal community composition. NZ, leaf spread of NT35 treatment; NJ, Fruit of NT35 treatment; NP, Root expansion of NT35 treatment; CKZ, leaf spread of control; CKJ, Fruit of control; CKP, Root expansion of control.

The dominant bacterial phyla were Myxococcota, Crenarchaeota, Chloroflexi, Actinobacteria, Verrucomicrobiota, and Proteobacteria, and the dominant fungal phyla were Ascomycota, Basidiomycota and Mortierellomycota. During the three stages of ginseng growth, there were differences in the proportions of the dominant microbial groups at the phylum level between the treatment with strain NT35 and the negative control and between the different growth stages under the same treatment (Figure 7).

In terms of the genus-level composition of the bacterial microbial communities (Figure 7A), *Mycobacterium*, *Luteimonas*, *Ellin6055*, *Nocardioides*, *Sphingomonas*, *Galella* and *Gemmatimonas* were the dominant genera in the rhizosphere soil treated with *B. velezensis* NT35 during the three stages of ginseng growth. For the control, the dominant genera consisted of *Candidatus solibacter*, *C. xiphinematobacter*, *C. nitrosotalea*, *C. udaeobacter*, RB41, *Aeromicrobium*, *Bradyrhizobium*, *Massilia*, *Rhodanobacter*, *Luteibacter*, *Pseudolabrys*, *Luteibacter* and *Massillia*. However, the abundances of some microorganisms also decreased. Without the NT35 treatment, the abundances of *Sphingomonas*, *Nocardioides*, *Ellin6055*, *Luteimonas*, *Galella*, *Mycobacterium* and *Jatrophihabitans* were always low. Additionally, the abundances of *Candidatus Solibacter*, *C. xiphinematobacter*, *C. nitrosotalea*, *C. udaeobacter*, *Bradyrhizobium*, *Pseudolabrys* and *Rhodanobacter* in the rhizosphere soil decreased significantly after treatment with the biocontrol bacterial strain NT35. For the genus composition of the fungal microbial community (Figure 7B), the fungal community treated without NT35 was composed mainly of *Fusarium*, *Neonectria*, *Thelonectria*, *Geomyces*, *Ilyonectria*, *Dactylonectria*, *Chaetomiun*, *Trichoderma*, and *Botryotrichum*. After treatment with *B. velezensis* NT35, *Penicillium*, *Pseudogymnoascus*, *Epicoccum*, *unidentified Sodariales* sp., *Cercophora*, *Cephalotrichum*, *Mortierella*, *Aspergillus*, *Apodus*, *Saitozyma* and *Trichoderma* became the dominant genera.

#### 3.9.3 Correlation analysis between the microbial community, soil enzymes and ginseng growth

**Figure 8:**
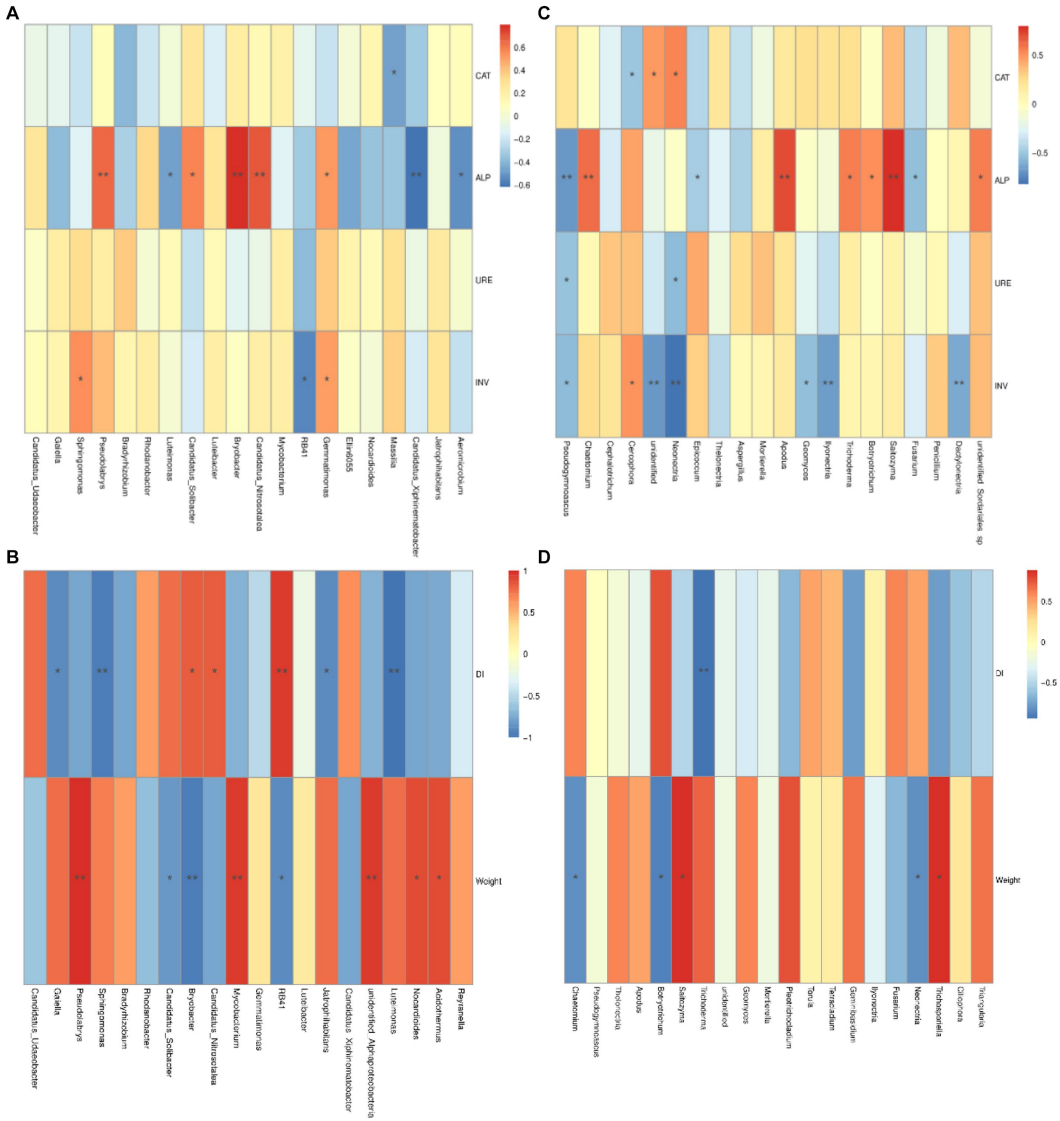
Associations between bacterial and fungal communities and relative factors. Correlation among bacterial microbial community and soil enzyme activity **(A)**, and disease index and root growth of ginseng **(B)**; Correlation among fungal microbial community and soil enzyme activity **(C)**; Disease index and root growth of ginseng **(D)**. CAT, catalase; ALP, alkaline phosphatase; URE, urease; INV, invertase. DI, disease index of ginseng rusty root rot; RL, root length of ginseng; RD, root diameter of ginseng. *p*-values were indicated by * symbol: ^**^p < 0.01; ^*^p < 0.05.

The correlation between the bacterial community composition and the four soil enzymes activity was shown in Figure 8A. There was a significant negative correlation between the abundance of the genus *Massilia* and catalase activity. The alkaline phosphatase activity was significantly negatively related to the abundances of *Aeromicrobium*, *Candidatus*_*xiphinematobacter* and *Luteimonas* and was significantly positively related to the abundances of *Gemmatimonas*, *C. solibacter*, *C. nitrosotalea*, *Bryobacter*, and *Pseudolabrys*. The invertase activity was significantly negatively related to the abundance of RB41 but significantly positively related to the abundances of *Gemmatimonas* and *Sphigomonas*.

On the other hand, the correlations between fungal community composition at the genus level and the activities of the four soil enzymes are shown in Figure 8C. The abundance of the genus *Cercophora* was significantly negatively correlated with catalase activity, and that of *Neonectria* was significantly positively correlated with catalase activity. Alkaline phosphatase activity was significantly negatively related to the abundances of *Fusarium* and *Epicoccum* and significantly positively related to the abundances of *Botryotrichia, Trichoderma*, *Saitozyma*, *Apodus* and *Chaetomium*. The abundances of *Neonectria* and *Pseudogymnoascus* were significantly negatively related to urease activity. Invertase activity was extremely significantly negatively related to the abundances of *Dacylonectria*, *Ilyonectria* and *Neonectria*; significantly negatively related to the abundances of *Geomyces* and *Pseudogymnoascus*; and significantly positively related to the abundance of the genus *Cercophora*.

The correlations between the microbial community at the genus level and the disease indices of rusty root rot and root growth in ginseng and bacterial composition are shown in Figure 8B. The abundances of *Acidothermus*, *Nocardioides*, *Bradyrhizobium*, *Sphingominas*, and *Psedolabrys* were significantly negatively related to the disease index of ginseng and were extremely significantly negatively related to *Gaiella*, *Jatrophihabitans* and *Luteimonas*, while the abundances of RB41 and *Candidatus udaeobacter* were significantly positively related to the disease index of ginseng rusty root rot. In terms of abundance, the length of the roots of the ginseng plants was strongly negatively related to *Acidothermus*, *Mycobacterium* and *Psedolabrys*; significantly negatively related to *Jatrophihabitans* and *Gaiella*; and significantly positively related to *C. nitrosotalea*, *Bryobacter* and *C. xiphinematobacter*. For the fungal community (Figures 8C,D), the disease index associated with ginseng rusty root rot was significantly negatively related to the abundance of *Geminibasidium* and *Trichoderma*. In terms of abundance, the ginseng root length was strongly negatively related to *Trichosporiella* and significantly negatively related to *Pleotrichocladium* but was extremely significantly positively related to *Botryotrichum*. The ginseng taproot diameter was strongly negatively related to the abundance of *Torula* and significantly negatively related to the abundances of *Tetracladium* and *Thelonectria*.

#### 3.9.4 LEfSe analysis of rhizosphere soil

**Figure 9:**
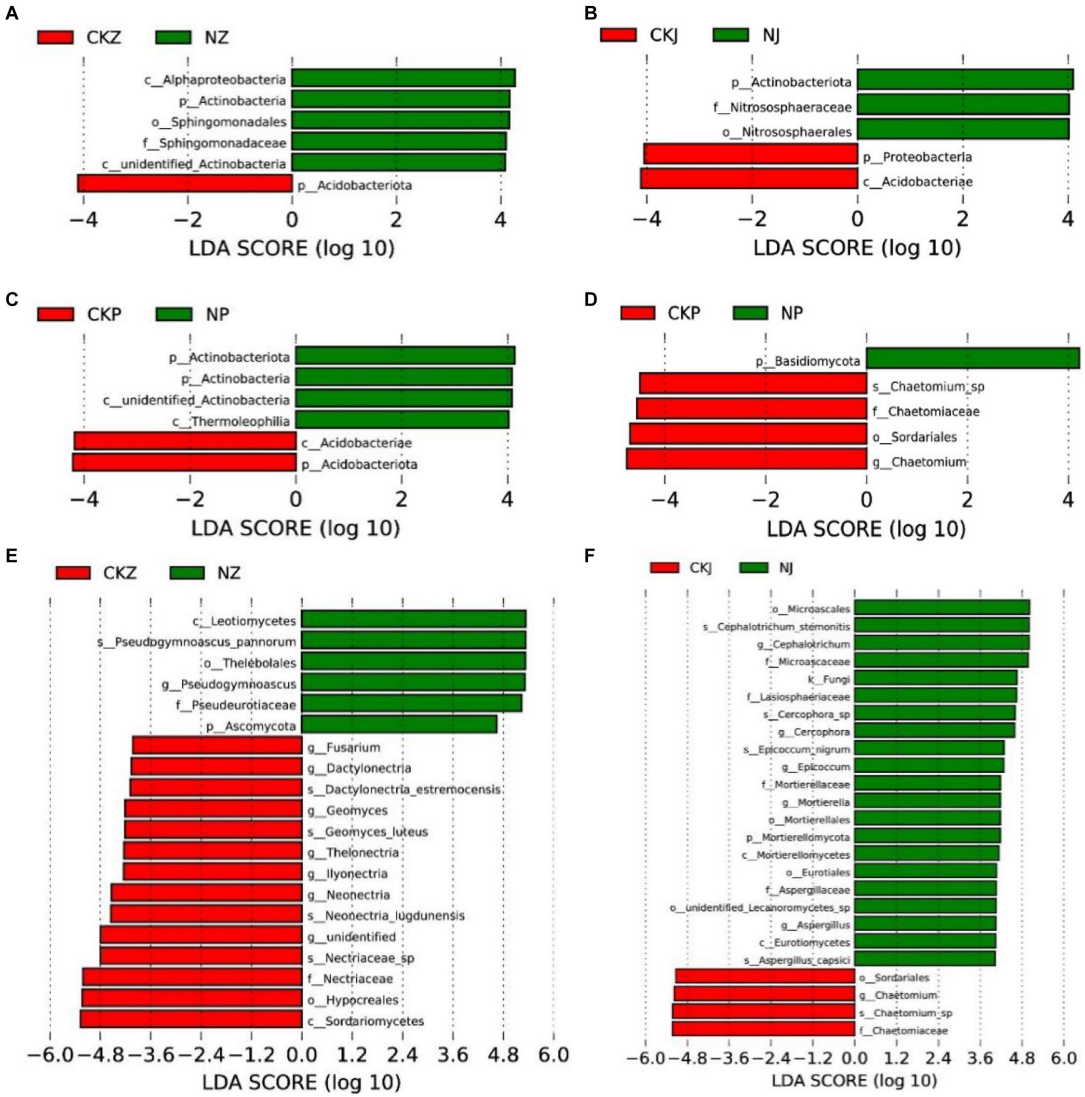
Linear discriminant effect size identified differentially abundant taxa of different periods of NT35 and control treatment (LDA significance threshold of 4.0). Bacterial community at the period of leaf spread **(A)**, fruit **(B)**, and root-expansion **(C)**; Fungal community at the period of leaf spread **(E)**, fruit **(F)**, and root expansion **(D)**. NZ, leaf-spread of NT35 treatment; NJ, fruit of NT35 treatment; NP, root expansion of NT35 treatment; CKZ, leaf-spreading of control; CKJ, fruiting of control; CKP, root expansion of control. The letters of k, p, c, o, f, g, and s were used to represent the kingdom, phylum, class, order, family, genus, and species, respectively.

The analysis of the bacterial community LEfSe in rhizosphere soil showed that *Acidobacteriota* was enriched in the soil without NT35 treatment, and *Alphaproteobacteria*, *Actinobacteria*, *Sphingomonadales* and *Sphingomonadaceae* were enriched in the NT35 treatment during the leaf-spreading period (Figure 9A). During the fruiting period, *Proteobacteria* and *Acidobacteria* were enriched in the control group, and in the NT35 treatment*, Actinobacteriota, Nitrososphaeraceae* and *Nitrososphaerales* were enriched (Figure 9B). During the root expansion stage, *Acidobacteria* and *Acidobacteriota* were enriched in the control group, and *Actinobacteriota*, *Actinobacteria* and *Thermoleophilia* were enriched in the NT35 treatment (Figure 9C).

According to the results of the fungal community LEfSe analysis, the soil not subjected to biocontrol bacterial treatment was enriched in *Fusarium*, *Dactylonectria*, *Dactylonectria*_*estremocensis*, *Geomyces*, *Geomyces*_*luteus*, *Thelonectria*, *Ilyonectria*, *Neonectria*, *Neonectria*_*lugdunensis*, *Nectriaceae*_sp., *Nectriaceae*, *Hypocreales* and *Sordariomycetes*. However, *Leotiomycetes*, *Pseudogymnoascus*_*pannorum*, *Thelebolales*, *Psedugymnascus*, *Pseudeurotiaceae* and *Ascomycota* were enriched after the NT35 treatment during the leaf-spreading stage (Figure 9E). There was enrichment of *Sordariales*, *Chaetomium*, *Chaetomium*_sp., and *Chaetomiaceae* in the control treatment at the fruiting stage, while the fungi *Microascales*, *Cephalotrichum*_*stemonitis*, *Cephalotrichum*, *Microascaceae*, *Lasiosphaeriaceae*, *Cercophora*, *Epicoccum*_*nigrum*, *Epicoccum*, *Mortierellaceae*, *Mortierella*, *Mortierellales*, *Mortierellomycota*, *Mortierellomycetes*, *Eurotiales*, *Aspergillaceae*, *Aspergillus*, *Eurotiomycetes* and *Aspergillus*_*capsici* were enriched in the NT35 treatment (Figure 9F). During the root expansion stage, the control group was enriched in *Chaetomium*_sp., *Chaetomiaceae*, *Sordariales* and *Chaetomium*, and *Basidiomycota* was enriched in the NT35 treatment (Figure 9D).

## 4 Discussion

In nature, *Bacillus* species exist widely and can be isolated from plant stems, leaves, roots and soil (Dinesh et al., 2015; Ali et al., 2020). Many articles have reported that *B. velezensis* can inhibit plant pathogens and be used as a biocontrol agent (Peng et al., 2019; Chen et al., 2020; Mta et al., 2020). In the field of biological control, research on screening antagonistic strains and control mechanisms is ongoing (Dimkić et al., 2022). The biocontrol bacterium *B. velezensis* strain NT35 was isolated from the rhizosphere soil of *P. ginseng*. In this study, the mechanism by which the *B. velezensis* strain NT35 controls ginseng rusty root rot was clarified by determining antagonistic effects and improvements in soil enzymes and microbial communities; however, these effects have rarely been described before in the field of controlling ginseng disease. Disease biocontrol mechanisms can be summarized as competition, antagonism and induction of plant resistance (Márquez et al., 2020; Ribeiro et al., 2021). The NT35 strain had a significant antagonistic effect on the mycelial growth, sporulation and spore germination of *I. robusta*, which causes ginseng rusty root rot.

The defense enzymes can increase plant resistance to biological stress and abiotic stress. *PAL* can prevent pathogen infection in the cell walls of host plants, and *CHI* combined with *β-1,3-GA* can induce a plant defense response to inhibit the growth of filamentous pathogenic fungi. *SOD* and *CAT* can eliminate endogenous free radicals to prevent cell death, which results in free radicals maintaining a normal dynamic level in plants to improve plant stress resistance (Manikandan et al., 2021; Wang Q. et al., 2023). For example, *B. velezensis* YYC induces an increase in the enzyme activity of *PAL*, *POD*, and *SOD* in tomato plants, resulting in resistance to bacterial wilt (Yan et al., 2022). Strawberry and grape plants can also obtain systemic resistance to external pathogens by increasing defense enzyme activity (Apaliya et al., 2017; Tang et al., 2022). Ginseng can also resist the invasion of pathogens by increasing the expression of defense-related enzyme-encoding genes (Em et al., 2020). The NT35 strain could significantly increase the expression of the *PAL*, *β-1,3-GA*, *CHI*, *SOD* and *CAT* genes in ginseng roots to increase systemic resistance to ginseng rusty root rot, and the control effect reached 51.99%.

In recent years, research on the rhizospheric soil microbial community structure has become a popular topic in the research of biological control mechanisms. The role of biocontrol bacteria in the early stage of microecology was mainly to cause microbial community changes in plant rhizosphere soil. The *B. velezensis* strain NT35 can change the alpha and beta diversity of the microbial community in ginseng rhizosphere soil, especially the Shannon and Simpson indices, according to PCoA. Notably, the NT35 strain can recruit several beneficial bacteria, such as *Luteimonas*, *Nocardioides*, *Sphingomonas*, and *Gemmatimonas,* from the rhizosphere soil. *Gemmatimonas* contains chlorophyll phototrophic species that can support plant life processes (Chen et al., 2022). Isolates of *Luteimonas* are associated with plant growth promotion and plant resistance to pathogens and have shown antifungal activity against wheat scab, wood brown rot and ash seedling pathogens (Xiao et al., 2017; Ulrich et al., 2022). *Nocardioides* can repair pesticides or other chemically contaminated soils (Benedek et al., 2022). *Sphingomonas* can metabolize prothioconazole and has a soil remediation function (Huang Z. et al., 2022). However, in the soil not treated with strain NT35, the enriched fungal community included *Fusarium*, *Neonectria*, *Ilyonectria*, and *Dactylonectria*, which cause ginseng root rot and rusty root rot (Farh et al., 2020; Fang et al., 2022; Walsh et al., 2022). *Ilyonectria robusta* is the main pathogenic fungus of ginseng studied in this paper. The relative abundance of *Ilyonectria* significantly decreased in the rhizosphere soil after the NT35 treatment. We also found that NT35 not only reduced the abundance of *Ilyonectria* in ginseng rhizosphere soil but also reduced the abundance of *Fusarium,* which causes root rot in ginseng plants. These results suggested that the NT35 strain has a strong biocontrol effect on ginseng rusty root rot. There is evidence that the introduction of biocontrol agents can increased soil enzyme activity and improved soil nutrient, provide a suitable environment for microorganisms in soil, generate a signature microbial composition (Tian et al., 2022). In addition, it has been reported that plants alter the rhizosphere microbial composition through root secretions (Lu et al., 2018). Here we also speculate that the strain NT35 improved soil nutritional conditions and might stimulate the formation of a special secretion pattern from ginseng root which recruit the beneficial microbes to affect the composition and structure of the rhizospheric soil microbial community.

The microbial community structure is affected by biological or abiotic factors, such as the seasonal climate, soil physical and chemical properties, microtopography, and plant activities, which affect the production of enzymes and enzyme reaction kinetics in soil (Bell et al., 2014). There was a close relationship between the microbial community and soil enzyme activity and plant roots (Bell et al., 2014). However, there have been few reports about the relationships among the disease indices of ginseng rusty root rot, root growth, soil enzyme activity and the microbial community after treatment with biocontrol agent. Among the four soil enzyme activities in this study, the bacterial community was strongly correlated with ALP activity, and the fungal community was highly correlated with both ALP and INV activity. However, the abundances of *Ilyonectria* and *Fusarium* were significantly negatively correlated with INV activity, indicating that INV activity could significantly affect the abundance of pathogenic fungi in ginseng rhizosphere soil (Figure 8C).

The effects on the disease index and root length were influenced mainly by the bacterial community. For example, the disease index was significantly negatively correlated with the abundance of *Sphingomonas,* which increased after the addition of strain NT35 (Figure 8B). The fungal community represented by *Trichoderma* was similar to that represented by *Sphingomonas* (Figure 8D). Therefore, the treatment with the *B. velezensis* strain NT35 can affect the rhizosphere microbial community, thereby reducing the disease index of ginseng rusty root rot. The effects of the biocontrol agent NT35 on ginseng rhizosphere soil are summarized as follows. First, strain NT35 can recruit beneficial bacteria and fungi, which not only have biocontrol functions but also degrade pesticides or even improve soil. Second, NT35 treatment can directly reduce the relative abundance of target pathogens in soil and control soil-borne diseases. Finally, the NT35 strain increased the correlations among soil enzyme activity, disease indices and microbial communities. LEfSe analysis revealed that the bacteria beneficial to plants, such as *Sphingomonadales* and *Sphingomonadaceae,* were enriched in the rhizosphere soil (Levy et al., 2018). Additionally, the NT35 treatment significantly enriched some actinomycetes that can secrete secondary metabolites to inhibit pathogens. The abundance of pathogenic fungi in the treatment inoculated with NT35 was significantly lower than that in the noninoculated NT35 treatment (Figure 9E).

## Data availability statement

The original contributions presented in the study are included in the article/Supplementary material, further inquiries can be directed to the corresponding authors.

## Author contributions

XL: Writing – original draft, Methodology, Investigation, Formal analysis, Data curation. JW: Writing – original draft, Methodology, Investigation, Formal analysis, Data curation. HS: Writing – original draft, Methodology, Investigation, Data curation. CX: Writing – original draft, Methodology, Data curation. LK: Writing – original draft, Methodology. YS: Writing – original draft, Data curation. WH: Writing – original draft, Resources. JG: Writing – review & editing, Resources, Project administration, Conceptualization. YJ: Writing – review & editing, Resources, Project administration, Funding acquisition, Conceptualization. CC: Writing – review & editing, Writing – original draft, Supervision, Project administration, Funding acquisition, Conceptualization.

## Footnotes

^1^http://www.arb-silva.de

## References

[ref2] Ahkami A. H. Allen White R. Handakumbura P. P. Jansson C. (2017). Rhizosphere engineering: enhancing sustainable plant ecosystem productivity. Rhizosphere. 3, 233–243. doi: 10.1016/j.rhisph.2017.04.012

[ref3] Ait Bessai S. Bensidhoum L. Nabti E. (2022). Optimization of IAA production by telluric bacteria isolated from northern Algeria. Biocatal. Agric. Biotechnol. 41:102319. doi: 10.1016/j.bcab.2022.102319

[ref4] Ali S. Hameed S. Shahid M. Iqbal M. Lazarovits G. Imran A. (2020). Functional characterization of potential PGPR exhibiting broad-spectrum antifungal activity. Microbiol. Res. 232:126389. doi: 10.1016/j.micres.2019.126389, PMID: 31821969

[ref5] Apaliya M. T. Zhang H. Yang Q. Zheng X. Zhao L. Kwaw E. (2017). *Hanseniaspora uvarum* enhanced with trehalose induced defense-related enzyme activities and relative genes expression levels against aspergillus tubingensis in table grapes. Postharvest Biol. Technol. 132, 162–170. doi: 10.1016/j.postharvbio.2017.06.008

[ref6] Bell C. W. Tissue D. T. Loik M. E. Wallenstein M. D. Zak J. C. (2014). Soil microbial and nutrient responses to 7 years of seasonally altered precipitation in a Chihuahuan Desert grassland. Glob. Chang. Biol. 20, 1657–1673. doi: 10.1111/gcb.12418, PMID: 24115607

[ref7] Benedek T. Pápai M. Gharieb K. Bedics A. Táncsics A. Tóth E.. (2022). Nocardioides carbamazepini sp. nov., an ibuprofen degrader isolated from a biofilm bacterial community enriched on carbamazepine. Syst. Appl. Microbiol. 45:126339. doi: 10.1016/j.syapm.2022.126339, PMID: 35714383

[ref8] Bhusal B. Mmbaga M. T. (2020). Biological control of Phytophthora blight and growth promotion in sweet pepper by Bacillus species. Biol. Control 150:104373. doi: 10.1016/j.biocontrol.2020.104373

[ref9] Chen M. Author W. N. C. Author L. F. C. Zhu Y. Chen Z. (2020). Biocontrol of tomato bacterial wilt by the new strain *Bacillus velezensis* FJAT-46737 and its lipopeptides. BMC Microbiol. 20, 160–112. doi: 10.1186/s12866-020-01851-2, PMID: 32539679 PMC7296739

[ref10] Chen L. Zhao Z. Li J. Wang H. Guo G. Wu W. (2022). Effects of muddy water irrigation with different sediment particle sizes and sediment concentrations on soil microbial communities in the Yellow River Basin of China. Agric Water Manag 270:107750. doi: 10.1016/j.agwat.2022.107750

[ref11] Cohen Y. Eyal H. Hanania J. (1989). Ultrastructure of Pseudoperonospora cubensis, in muskmelon genotypes susceptible and resistant to downy mildew. Physiol. Mol. Plant Pathol. 34, 27–40. doi: 10.1016/0885-5765(89)90014-3

[ref12] Delaporte-Quintana P. Lovaisa C. N. Rapisarda A. V. Pedraza R. O. (2020). The plant growth promoting bacteria Gluconacetobacter diazotrophicus and *Azospirillum brasilense* contribute to the iron nutrition of strawberry plants through siderophores production. Plant Growth Regul. 91, 185–199. doi: 10.1007/s10725-020-00598-0

[ref13] Dimkić I. Janakiev T. Petrović M. Degrassi G. Fira D. (2022). Plant-associated Bacillus and Pseudomonas antimicrobial activities in plant disease suppression via biological control mechanisms—a review. Physiol. Mol. Plant Pathol. 117:101754. doi: 10.1016/j.pmpp.2021.101754

[ref14] Dinesh R. Anandaraj M. Kumar A. Bini Y. K. Subila K. P. Aravind R. (2015). Isolation, characterization, and evaluation of multi-trait plant growth promoting rhizobacteria for their growth promoting and disease suppressing effects on ginger. Microbiol. Res. 173, 34–43. doi: 10.1016/j.micres.2015.01.014, PMID: 25801969

[ref15] Edgar R. C. (2013). UPARSE: highly accurate OTU sequences from microbial amplicon reads. Nat. Methods 10, 996–998. doi: 10.1038/nmeth.2604, PMID: 23955772

[ref16] Em A. Tat B. Kah A. (2020). Role of plant-growth promoting fungi (PGPF) in defensive genes expression of *Triticum aestivum* against wilt disease. Rhizosphere. 15:100223. doi: 10.1016/j.rhisph.2020.100223

[ref17] Fang W. Liu X. Song Z. Jin X. Yan D. Wang Q.. (2022). Mechanism of the antifungal action of chloropicrin fumigation against Panax notoginseng root rot caused by fusarium solani. Physiol. Mol. Plant Pathol. 121:101859. doi: 10.1016/j.pmpp.2022.101859

[ref18] Farh M. E.-A. Kim Y.-J. Abbai R. Singh P. Jung K.-H. Kim Y.-J.. (2020). Pathogenesis strategies and regulation of ginsenosides by two species of Ilyonectria in *Panax ginseng*: power of speciation. J. Ginseng Res. 44, 332–340. doi: 10.1016/j.jgr.2019.02.001, PMID: 32148416 PMC7031752

[ref19] Farh M. E.-A. Kim Y.-J. Kim Y.-J. Yang D.-C. (2018). Cylindrocarpon destructans/Ilyonectria radicicola-species complex: causative agent of ginseng root-rot disease and rusty symptoms. J. Ginseng Res. 42, 9–15. doi: 10.1016/j.jgr.2017.01.004, PMID: 29348716 PMC5766697

[ref20] Fu G. Huang S. Ye Y. Wu Y. Cen Z. Lin S. (2010). Characterization of a bacterial biocontrol strain B106 and its efficacies on controlling banana leaf spot and post-harvest anthracnose diseases. Biol. Control 55, 1–10. doi: 10.1016/j.biocontrol.2010.05.001

[ref21] Guan Y. M. Ma Y. Y. Jin Q. Wang Q. X. Liu N. Fu Y. P.. (2020). Multi-locus phylogeny and taxonomy of the fungal complex associated with rusty root rot of *Panax ginseng* in China. Front. Microbiol. 11:618942. doi: 10.3389/fmicb.2020.618942, PMID: 33391250 PMC7772391

[ref22] Huang Z. Li Q. Gai X. Zhang X. Zhong Z. Bian F.. (2022). Corrigendum: effects of on-and off-year management practices on the soil organic C fractions and microbial community in a Moso bamboo (*Phyllostachys edulis*) forest in subtropical China. Front. Plant Sci. 13:1020344. doi: 10.3389/fpls.2022.1020344, PMID: 36570912 PMC9780457

[ref23] Huang J. Li M. Jin F. Wang Z. Li W. Pan D.. (2022). Isolation of Sphingomonas sp. AJ-1 and its enantioselective S-methylation of the triazole fungicide prothioconazole. Sci. Total Environ. 851:158220. doi: 10.1016/j.scitotenv.2022.158220, PMID: 36007644

[ref24] Iyer B. Rajput S. M. Rajkumar S. (2017). Effect of succinate on phosphate solubilization in nitrogen fixing bacteria harbouring chick pea and their effect on plant growth. Microbiol. Res. 202, 43–50. doi: 10.1016/j.micres.2017.05.005, PMID: 28647122

[ref25] Jiang Y. Song Y. Jiang C. Y. Li X. Liu T. T. Wang J.. (2022). Identification and characterization of *Arthrobacter nicotinovorans* JI39, a novel plant growth-promoting Rhizobacteria strain from *Panax ginseng*. Front. Plant Sci. 13:873621. doi: 10.3389/fpls.2022.873621, PMID: 35615118 PMC9125309

[ref26] Kandeler E. Gerber H. (1988). Short-term assay of soil urease activity using colorimetric determination of ammonium. Biol. Fertil. Soils 6, 68–72. doi: 10.1007/BF00257924

[ref27] Kim Y. Lim J. Sukweenadhi J. Seok W. J. Lee S. W. Park J. C.. (2019). Genomic characterization of a newly isolated Rhizobacteria Sphingomonas panacis reveals plant growth promoting effect to Rice. Biotechnol. Bioprocess Eng. 24, 119–125. doi: 10.1007/s12257-018-0386-2

[ref28] Lee J.-H. Lee J.-S. Kwon W.-S. Kang J.-Y. Lee D.-Y. In J.-G.. (2015). Characteristics of Korean ginseng varieties of Gumpoong, Sunun, Sunpoong, Sunone, Cheongsun, and Sunhyang. J. Ginseng Res. 39, 94–104. doi: 10.1016/j.jgr.2014.06.007, PMID: 26045682 PMC4452533

[ref29] Levy A. Salas G. I. Mittelviefhaus M. Clingenpeel S. Herrera P. S. Miao J.. (2018). Genomic features of bacterial adaptation to plants. Nat. Genet. 50, 138–150. doi: 10.1038/s41588-017-0012-9, PMID: 29255260 PMC5957079

[ref30] Linden C. Fatouros N. E. Kammenga J. E. (2022). The potential of entomopathogenic nematodes to control moth pests of ornamental plantings. Biol. Control 165:104815. doi: 10.1016/j.biocontrol.2021.104815

[ref31] Liu X. Y. Chen C. Y. Wang J. Zhou S. H. Long X. X. (2021). Phosphorus solubilizing bacteria Bacillus thuringiensis and *Pantoea ananatis* simultaneously promote soil inorganic phosphate dissolution and soil Pb immobilization. Rhizosphere 20, 100448–102198. doi: 10.1016/j.rhisph.2021.100448

[ref32] Livak K. J. Schmittgen T. D. (2001). Analysis of relative gene expression data using real-time quantitative PCR and the 2^−ΔΔCt^ method. Methods 25, 402–408. doi: 10.1006/meth.2001.1262 11846609

[ref33] Lu T. Ke M. J. Lavoie M. Jin Y. J. Fan X. J. Zhang Z. Y.. (2018). Rhizosphere microorganisms can influence the timing of plant flowering. Microbiome 6, 1–12. doi: 10.1186/s40168-018-0615-0 30587246 PMC6307273

[ref34] Lúcia R. R. B. Newton P. S. Lilia G. W. Delson L. Marcos A. B. L. Samuel M. M. M.. (2016). Cowpea resistance induced against fusarium oxysporum f. sp. tracheiphilum by crustaceous chitosan and by biomass and chitosan obtained from Cunninghamella elegans. Biol. Control 92, 45–54. doi: 10.1016/j.biocontrol.2015.09.006

[ref35] Manikandan A. Parthasarathy R. Anusuya S. Huang J. (2021). An overview of plant defense-related enzymes responses to biotic stresses. Plant Gene. 27:100302. doi: 10.1016/j.plgene.2021.100302

[ref36] Márquez R. Blanco E. L. Aranguren Y. (2020). Bacillus strain selection with plant growth-promoting mechanisms as potential elicitors of systemic resistance to gray mold in pepper plants. Saudi J Biol Sci. 27, 1913–1922. doi: 10.1016/j.sjbs.2020.06.015, PMID: 32714014 PMC7376110

[ref37] Mta B. Ila B. Bt C. Lt C. Isa B. Vba B. (2020). Growth promotion on horticultural crops and antifungal activity of *Bacillus velezensis* XT1. Appl. Soil Ecol. 150:103453. doi: 10.1016/j.apsoil.2019.103453

[ref38] Niemi R. M. Heiskanen I. Wallenius K. Lindström K. (2001). Extraction and purification of DNA in rhizosphere soil samples for PCR-DGGE analysis of bacterial consortia. J. Microbiol. Methods 45, 155–165. doi: 10.1016/S0167-7012(01)00253-6, PMID: 11348673

[ref39] Ning Z. Y. Li Y. L. Zhao X. Y. Han D. Zhan J. (2022). Comparison of leaf and fine root traits between annuals and perennials, implicating the mechanism of species changes in desertified grasslands. Front. Plant Sci. 12:778547. doi: 10.3389/fpls.2021.778547, PMID: 35185947 PMC8854787

[ref40] Peng G. Zhao X. Y. Li Y. Z. Wang R. Huang Y. Qi G. F. (2019). Engineering *Bacillus velezensis* with high production of acetoin primes strong induced systemic resistance in *Arabidopsis thaliana*. Microbiol. Res. 227:126297. doi: 10.1016/j.micres.2019.126297 31421711

[ref41] Quast C. Pruesse E. Yilmaz P. Gerken J. Schweer T. Yarza P.. (2013). The SILVA ribosomal RNA gene database project: improved data processing and web-based tools. Nucleic Acids Res. 41, D590–D596. doi: 10.1093/nar/gks1219, PMID: 23193283 PMC3531112

[ref42] Rahman M. Punja Z. K. (2005). Factors influencing development of root rot on ginseng caused by Cylindrocarpon destructans. Phytopathology 95, 1381–1390. doi: 10.1094/PHYTO-95-1381, PMID: 18943548

[ref43] Rahul J. Anita P. (2016). Soil enzymes and microbial endophytes as indicators of climate variation along an altitudinal gradient with respect to wheat rhizosphere under mountain ecosystem. Rhizosphere. 2, 75–84. doi: 10.1016/j.rhisph.2016.07.007

[ref44] Ribeiro I. Bach E. Moreira F. Müller A. Passaglia L. (2021). Antifungal potential against Sclerotinia sclerotiorum (lib.) de Bary and plant growth promoting abilities of Bacillus isolates from canola (*Brassica napus* L.) roots. Microbiol. Res. 248:126754. doi: 10.1016/j.micres.2021.126754, PMID: 33848783

[ref45] Sharma M. Manhas R. K. (2022). Biocontrol potential of Streptomyces sp. M4 and salvianolic acid B produced by it against Alternaria black leaf spot. Microb. Pathog. 173:105869. doi: 10.1016/j.micpath.2022.105869, PMID: 36356795

[ref46] Stoll A. Salvatierra-Martínez R. González M. Araya M. (2021). The role of Surfactin production by *Bacillus velezensis* on colonization, biofilm formation on tomato root and leaf surfaces and subsequent protection (ISR) against Botrytis cinerea. Microorganisms. 9:2251. doi: 10.3390/microorganisms9112251, PMID: 34835375 PMC8626045

[ref47] Sun X. D. Ye Y. Q. Ma Q. X. Guan Q. W. Jones D. L. (2021). Variation in enzyme activities involved in carbon and nitrogen cycling in rhizosphere and bulk soil after organic mulching. Rhizosphere. 19:100376. doi: 10.1016/j.rhisph.2021.100376

[ref48] Tang Y. Li R. Jiang Z. C. Cheng Z. Y. Li W. Shao Y. Z. (2022). Combined effect of Debaryomyces hansenii and *Bacillus atrophaeus* on the physicochemical attributes, defense-related enzyme activity, and transcriptomic profile of stored litchi fruit. Biol. Control 172:104975. doi: 10.1016/j.biocontrol.2022.104975

[ref49] Tian Y. Liu Y. Yue L. Uwaremwe C. Zhao X. Zhou Q.. (2022). Bacterial inoculant and sucrose amendments improve the growth of *Rheum palmatum* L. by reprograming its metabolite composition and altering its soil microbial community. Int. J. Mol. Sci. 23:1694. doi: 10.3390/ijms23031694, PMID: 35163617 PMC8835959

[ref50] Tiwari S. Sarangi B. K. Thul S. T. (2016). Identification of arsenic resistant endophytic bacteria from *Pteris vittata* roots and characterization for arsenic remediation application. J. Environ. Manage. 180, 359–365. doi: 10.1016/j.jenvman.2016.05.029, PMID: 27257820

[ref51] Ulrich K. Becker R. Behrendt U. Kube M. Schneck V. Ulrich A. (2022). Physiological and genomic characterisation of Luteimonas fraxinea sp. nov., a bacterial species associated with trees tolerant to ash dieback. Syst. Appl. Microbiol. 45:126333. doi: 10.1016/j.syapm.2022.126333, PMID: 35605315

[ref52] Walsh J. P. McMullin D. R. Yeung K. K. C. Sumarah M. W. (2022). Resorcylic acid lactones from the ginseng pathogen Ilyonectria mors-panacis. Phytochem. Lett. 48, 94–99. doi: 10.1016/j.phytol.2022.02.008

[ref53] Wang J. Wang J. R. Liu T. T. Li X. Gao J. Jiang Y.. (2023). *Bacillus amyloliquefaciens* FG14 as a potential biocontrol strain against rusty root rot of Panax ginseng, and its impact on the rhizosphere microbial community. Biol. Control 182:105221. doi: 10.1016/j.biocontrol.2023.105221

[ref54] Wang H. Xu F. Wang X. Wang X. Q. Kwon W. S. Yang D. C. (2019). Molecular discrimination of *Panax ginseng* cultivar K-1 using pathogenesis-related protein 5 gene. J. Ginseng Res. 43, 482–487. doi: 10.1016/j.jgr.2018.07.001, PMID: 31308820 PMC6606972

[ref55] Wang C. X. Zhao X. L. Wu K. Liang C. Y. Liu J. Yang H.. (2023). Isolation and characterization of *Bacillus velezensis* strain B19 for biocontrol of Panax notoginseng root rot. Biol. Control 185:105311. doi: 10.1016/j.biocontrol.2023.105311

[ref9001] Wang Q. Xu X. Y. Li X. H. Wang J. L. (2023). Soil enzyme activities, physiological indicators, agronomic traits and yield of common buckwheat under herbicide combined with safeners. Sci. Total Environ. 903:166261. doi: 10.1016/j.scitotenv.2023.166261 37579798

[ref56] Wołejko E. Jabłońska-Trypuć A. Wydro U. Butarewicz A. Łozowicka B. (2020). Soil biological activity as an indicator of soil pollution with pesticides – a review. Appl. Soil Ecol. 147:103356. doi: 10.1016/j.apsoil.2019.09.006

[ref57] Xiao X. Fan M. Wang E. Chen W. Wei G. (2017). Interactions of plant growth-promoting rhizobacteria and soil factors in two leguminous plants. Appl. Microbiol. Biotechnol. 101, 8485–8497. doi: 10.1007/s00253-017-8550-8, PMID: 29038972

[ref58] Xu Y. P. Zang R. C. Chen W. L. Lou Y. C. (2001). Effect of *Enterobacter cloacae* B8 fermentation broth on plant growth and analysis of IAA. J Zhejiang Univ. 49, 1370–1375. doi: 10.1360/04wc0011

[ref59] Yan Y. C. Xu W. H. Hu Y. L. Tian R. M. Wang Z. G. (2022). *Bacillus velezensis* YYC promotes tomato growth and induces resistance against bacterial wilt. Biol. Control 172:104977. doi: 10.1016/j.biocontrol.2022.104977

[ref60] You L. Cha S. H. Kim M.-Y. Cho J. Y. (2022). Ginsenosides are active ingredients in *Panax ginseng* with immunomodulatory properties from cellular to organismal levels. J. Ginseng Res. 46, 711–721. doi: 10.1016/j.jgr.2021.12.007, PMID: 36312737 PMC9597430

[ref61] Zhang J. Y. Ding X. Guan R. Zhu C. M. Xu C. Zhu B. C.. (2018). Evaluation of different 16S rRNA gene V regions for exploring bacterial diversity in a eutrophic freshwater lake. Sci. Total Environ. 618, 1254–1267. doi: 10.1016/j.scitotenv.2017.09.228, PMID: 29089134

[ref62] Zhang X. Y. Zhou Y. Li J. Gu X. Y. Zhao L. N. Li B.. (2022). Pichia caribbica improves disease resistance of cherry tomatoes by regulating ROS metabolism. Biol. Control 169:104870. doi: 10.1016/j.biocontrol.2022.104870

[ref63] Zhao W. Guo Q. Li S. Lu X. Y. Dong L. H. Wang P. P.. (2022). Application of *Bacillus subtilis* NCD-2 can suppress cotton verticillium wilt and its effect on abundant and rare microbial communities in rhizosphere. Biol. Control 165:104812. doi: 10.1016/j.biocontrol.2021.104812

