# Supplementary material for: Biocontrol and growth promotion potential of Bacillus velezensis NT35 on Panax ginseng based on the multifunctional effect
Source: Front Microbiol. 2024 Jul 30;15:1447488. doi: 10.3389/fmicb.2024.1447488 (PMC11319169; doi:10.3389/fmicb.2024.1447488)
Supplement: Supplementary file 1 [file Data_Sheet_1.pdf]

Table S1 Primers of real-time PCR for Real-time PCR detection of ginsenoside content

Gene	GenBank ID	Primer	Sequence（5’-3’）	Tm ℃
PgSS	AB115496	PgSS-F	CCTCGCCAGATTTGGAGTAA	62
		PgSS -R	GCACAGAACCGGAAGATAGC	
PgSE	GU323920	PgSS -F	GATGTGCCTGGACAAAAGGT	66
		PgSS -R	AGGATGGCGCGCATATTGAAAG	
PgDS	JN596111	PgSS -R	GAGAGATCCGACACCTCTGC	61
		PgSS -F	ATTTTGAGCTGCTGGTGCTT	
β-actin	AY907207	ACT-F	TGCCCCAGAAGAGCACCCTGT	62
		ACT-R	AGCATACAGGGAAAGATCGGCTTGA	

Table S2 Inhibitory rate of different concentrations of strain NT35 on *I. robusta*

Bacterial concentration ($CFU \cdot mL^{-1}$)	Fungal mycelia diameter (mm)	Inhibition rate (%)
CK	59.00±0.10	-
10^9	8.25±0.03	99.51±0.41%^{a*}
10^8	8.75±0.03	98.53±0.33%^{a}
10^7	11.50±0.15	94.12±2.29%^{a}
10^6	26.25±0.58	64.22±0.86%^{b}
10^5	26.50±0.35	63.73±0.62%^{b}
10^4	34.00±0.05	49.02±0.15%^{bc}
10^3	37.25±1.175	42.65±1.79%^{bc}
10^2	41.50±0.35	34.31±0.45%^{c}
10^1	43.00±0.20	31.37±0.23%^{c}

* Valuse represent mean ± standard deviation (n = 3).
* The same letters indicate no significant differences ($p \leq 0.05$).

Table S3 Inhibitory rate of strain NT35 on spore sporulation and germination of *I. robusta*

Concentration ($CFU \cdot mL^{-1}$)	Number of spores ($\times 10^5$)	Inhibition rate of sporulation (%)	Spore germination rate (%)	Inhibition rate of spore germination (%)
CK	133±3.07	100±0 ^{a}*	91.40±0.13	-
10^9	0±0	100±0 ^{a}	6.12±1.57	93.28±1.71 ^{a}
10^8	0±0	100±0 ^{a}	8.82±0.48	90.31±0.54 ^{a}
10^7	0±0	100±0 ^{a}	10.99±0.64	87.93±0.72 ^{b}
10^6	0±0	100±0 ^{a}	16.71±1.71	81.65±1.85 ^{c}
10^5	0±0	100±0 ^{a}	20.87±0.41	77.08±0.49 ^{d}
10^4	0±0	100±0 ^{a}	27.87±1.08	69.39±1.23 ^{e}
10^3	0.95±0.02	99.28±0.22 ^{b}	30.78±0.17	66.19±0.14 ^{e}
10^2	3.60±0.15	97.29±1.20 ^{c}	43.76±2.58	51.54±2.91 ^{f}
10^1	1.22±0.23	90.80±2.51 ^{d}	59.67±0.85	34.45±1.03 ^{g}

*Each value is the mean of three independent experiments.
*The same letters indicate no significant differences between means as determined by the least significant difference test ($p \leq 0.05$).

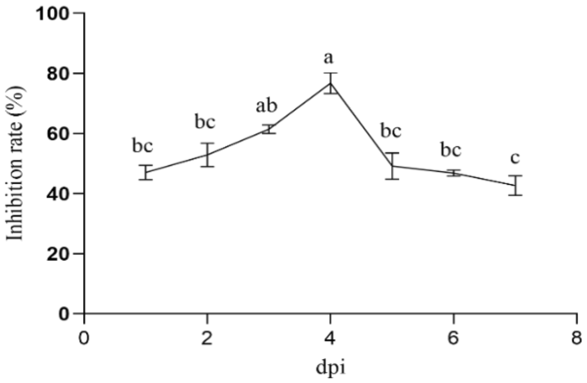

Figure S1 Inhibitory rate of NT35 on mycelium weight of *I. robusta*
Note: n = 3; different values (a, b, c) between different dpi are significantly different ($p < 0.05$).

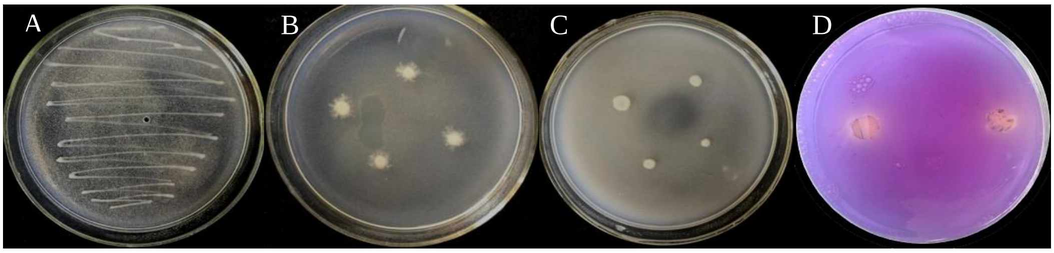

Figure S2 Growth-promoting shape of strain NT35
(A) Nitrogen fixation; (B) Potassium dissolving; (C) Phosphorus dissolving; D. Iron bearing

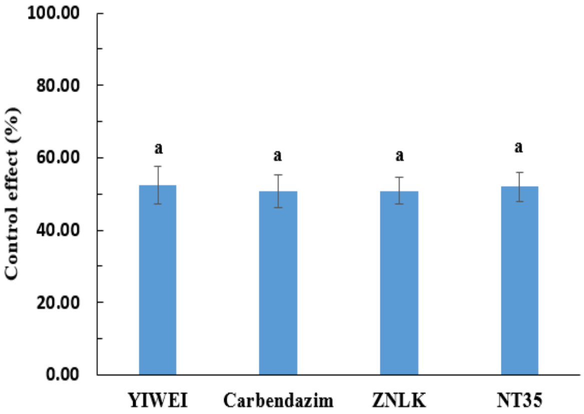

Figure S3 The control effect of NT35 on ginseng rusty root rot
Valuse represent mean ± standard deviation (n = 3), the same letters between different treatments indicate no significant differences ($p \leq 0.05$).

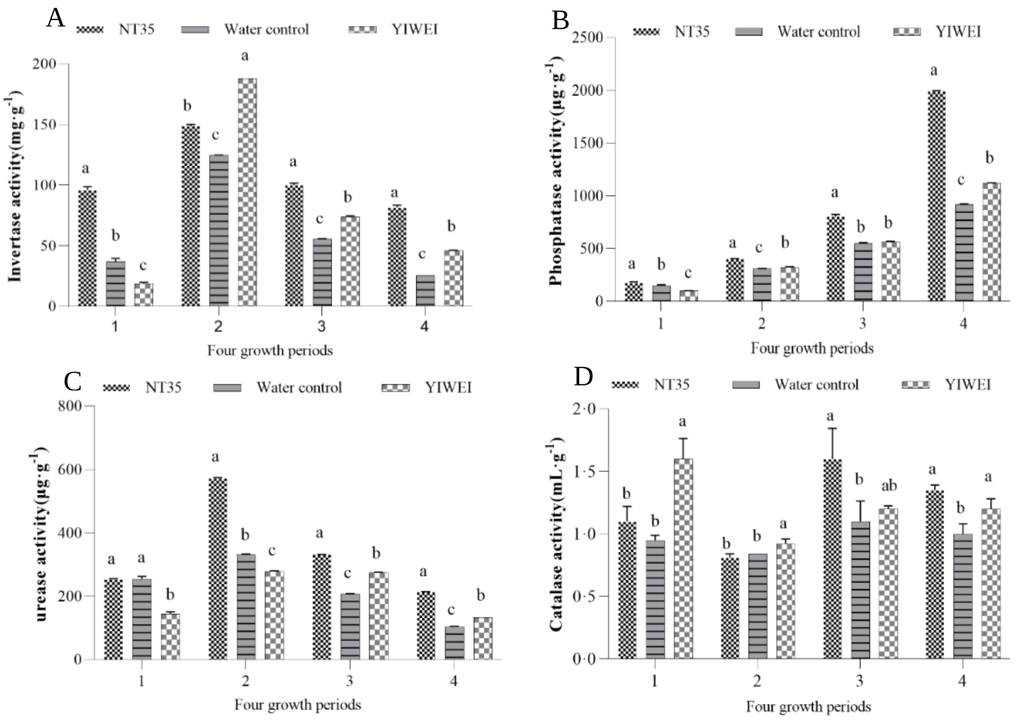

Figure S4 Effects of strain NT35 on ginseng soil enzyme activity at different periods
(A) invertase; (B) alkaline phosphatase; (C) urease; (D) catalase.
1. leaf spread stage; 2. fruit stage; 3. root expansion stage; 4. mature stage.
The error bars reflect standard deviations from n = 3 independent replicates, different values (a, b, c) are significantly different ($p < 0.05$).

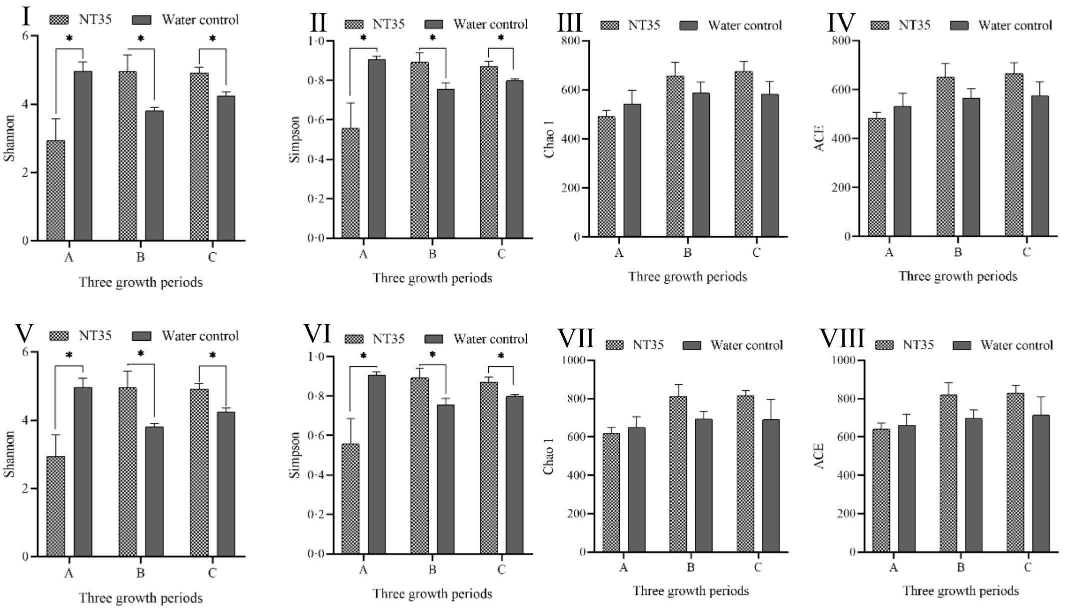

Figure S5 Diversity of bacteria and fungi in rhizosphere soil in different periods after strain NT35 treatment
(I) Shannon of bacterium; (II) Simpson of bacterium; (III) Chao 1 of bacterium; (IV) ACE of bacterium;
(V) Shannon of fungus; (VI) Simpson of fungus; (VII) Chao 1 of fungus; (VIII) ACE of fungus.
A：leaf spread stage B: Fruit stage C: Root expansion stage. Each value is the mean of three independent experiments, p-values were indicated by * symbol: **= $p < 0.01$; *=$p < 0.05$.

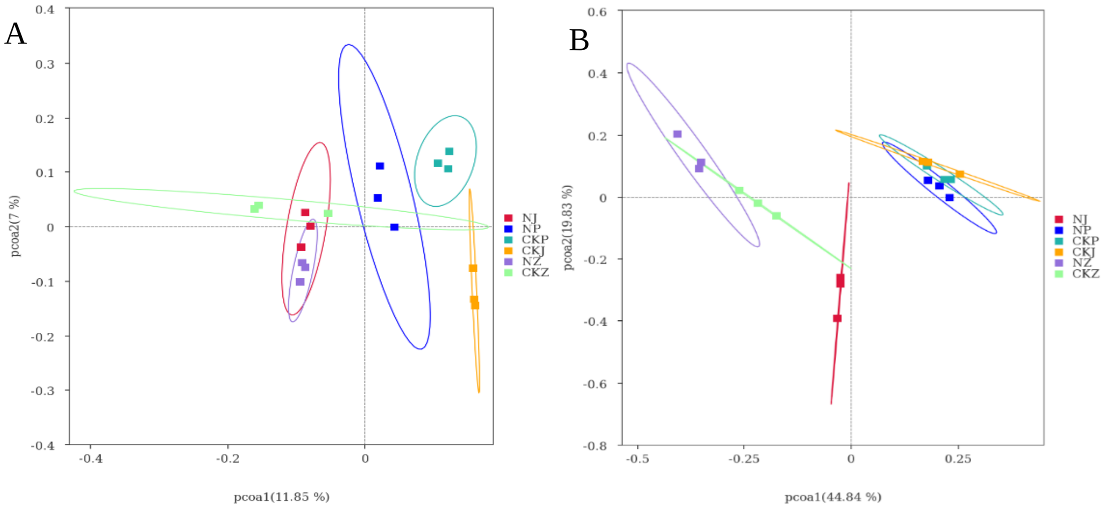

Figure S6 PCoA in microbial communities (OTUs) of soils under different treatments.
(A) Bacteria community; (B) Fungi community.
NZ: leaf spread of NT35 treatment; NJ: Fruit of NT35 treatment; NP: Root expansion of NT35 treatment;
CKZ: leaf spread of control; CKJ: Fruit of control; CKP: Root expansion of control.
